# African-specific genetic loci determine iron status and risk of severe malaria and bacteremia in African children

**DOI:** 10.1038/s41467-026-71567-w

**Published:** 2026-04-07

**Authors:** John Muthii Muriuki, Alexander J. Mentzer, Gavin Band, Amanda Y. Chong, Alex W. Macharia, Reagan M. Mogire, Kelvin Mokaya Abuga, Ruth Mitchell, James J. Gilchrist, Emily L. Webb, Francis M. Ndungu, Laura M. Raffield, Lynette Ekunwe, Amy R. Bentley, Sodiomon B. Sirima, Shabir A. Madhi, Adrian V. S. Hill, Andrew M. Prentice, Philip Bejon, Gibran Hemani, George Davey Smith, Manjinder S. Sandhu, Alison M. Elliott, Thomas N. Williams, Adebowale Adeyemo, Sarah H. Atkinson

**Affiliations:** 1 KEMRI-Wellcome Trust Research Programme, Kilifi, Kenya; 2 Centre for Human Genetics, University of Oxford, Oxford, UK; 3 CAMS Oxford Institute, University of Oxford, Oxford, UK; 4 National Human Genome Research Institute, National Institutes of Health, Bethesda, MD USA; 5 MRC Integrative Epidemiology Unit, University of Bristol, Bristol, UK; 6 Oxford Vaccine Group, University of Oxford, Oxford, UK; 7 MRC International Statistics and Epidemiology Group, London School of Hygiene & Tropical Medicine, London, UK; 8 Centre for Tropical Medicine and Global Health, University of Oxford, Oxford, UK; 9 Department of Genetics, University of North Carolina, Chapel Hill, NC USA; 10 Department of Medicine, University of MS Medical Center, Jackson, USA; 11 Groupe de Recherche Action en Sante (GRAS), Ouagadougou, Burkina Faso; 12 South African Medical Research Council Vaccines and Infectious Diseases Analytics Research Unit, University of the Witwatersrand, Johannesburg, South Africa; 13 The Jenner Institute, University of Oxford, Oxford, UK; 14 MRC Unit The Gambia at London School of Hygiene & Tropical Medicine, Banjul, The Gambia; 15 Modernising Medical Microbiology Unit, University of Oxford, Oxford, UK; 16 Department of Epidemiology & Biostatistics, Imperial College London, London, UK; 17 MRC/Uganda Virus Research Institute and London School of Hygiene & Tropical Medicine Uganda Research Unit, Entebbe, Uganda; 18 Department of Clinical Research, London School of Hygiene & Tropical Medicine, London, UK; 19 Department of Surgery and Cancer, Imperial College London, London, UK; 20 Department of Paediatrics, University of Oxford, Oxford, UK

**Keywords:** Genome-wide association studies, Predictive markers, Bacterial infection, Malaria

## Abstract

Iron is essential for both humans and pathogens, yet its genetic regulation remains understudied in African populations. Here, we report genome-wide association studies of six iron-related biomarkers in 3928 children from five sites across Africa, with replication in 2868 African American adults and investigate associations with severe malaria and bacteremia. We identify previously unreported loci at genome-wide significance, for transferrin at *GTF3C5*, and for hepcidin at *CHCHD7*/*SDR16C5*. Variants tagging the DUP4 haplotype, encoding the Dantu blood group (rs552439837) are associated with soluble transferrin receptor levels. Variants at *GTF3C5* (rs2905094) and DUP4 confer protection against severe malaria and bacteremia. The *CHCHD7*/*SDR16C5* variant (rs73596248) increases hepcidin levels and is associated with reduced risk of *Klebsiella pneumoniae* and *Staphylococcus aureus* bacteremia. Polygenic risk scores derived from European data show limited transferability to African populations. In this work, we demonstrate new genetic insights into iron regulation and highlight iron’s role in host-pathogen interactions.

## Abstract (web summary)

This genetic study of nearly 4,000 children identifies African specific variants that influence iron levels. These variants are also associated with risk of severe malaria and bacteremia, highlighting iron’s role in host-pathogen interactions.

## Introduction

Iron is an essential nutrient required for critical biological functions in both humans and pathogens. The evolutionary balance between maintaining adequate iron for physiological functions and restricting its availability to pathogens is especially delicate in African populations, due to the high burden of iron deficiency (ID) and infectious disease. ID affects over half of young children living in sub-Saharan Africa (SSA)^1^ accounting for up to 50% of anemia cases^2^. In contrast to other WHO regions, the prevalence of ID has been rising in SSA^3^, while malaria and bacterial infections remain as major causes of morbidity and mortality. In 2023, 95% of the estimated 263 million cases and 597,000 deaths from malaria globally occurred in the WHO African region^4^. Similarly, age-standardized mortality rates due to invasive bacterial infections were highest in sub-Saharan Africa in 2019^5^.

Iron plays a critical role in the relationship between host and pathogen^6^ and we hypothesize that infectious diseases, including malaria and bacterial infections, may have exerted powerful selective pressure on iron-regulating genes in Africa. Iron homeostasis is tightly regulated in humans since both iron deficiency and overload alter the risk of infection^7^. For instance, iron supplementation is associated with increased risk of malaria infection^8^, and ID protects against malaria infection in observational studies^9^. Iron is tightly sequestered by transferrin to reduce its availability to systemic organisms, while cellular iron uptake through transferrin receptor 1 is critical for the development of host immunity^10^. During inflammation and infection iron availability is further reduced by the actions of hepcidin which inhibits enteric absorption and sequesters plasma iron into macrophages^6,11^ thus inhibiting the growth of malaria parasites and bacteria^12,13^.

The genetic architecture of iron status in African ancestry populations remains understudied, in contrast to the many large-scale genome-wide association studies (GWAS) of iron status conducted in European ancestry populations^14,15^. Studies conducted in Africa are largely limited to a few candidate gene studies^16,17^ and to our knowledge, this is the first GWAS of iron biomarkers undertaken in continental African populations. A single GWAS of iron status has been conducted in middle-aged African-American adults^18^, however transferability to continental African populations might be complicated by admixture^19^, limited representation of the greater genetic diversity found among continental Africans^20,21^, and by different environmental exposures in terms of nutrition and infectious disease^22^. Even within Africa, divergent patterns in linkage disequilibrium might yield region-specific patterns of association^23^. Given the greater genetic diversity^20^ and selective pressure from malaria and other infections in continental African populations, an iron GWAS may reveal differential effects compared to other populations due to genotype-environment interactions.

In this work, to enhance discovery of genetic determinants of iron status in an understudied population, we conducted a GWAS of six iron biomarkers (transferrin, hepcidin, soluble transferrin receptor (sTfR), ferritin, serum iron, and transferrin saturation (TSAT)) in 3928 children recruited from Kenya, Uganda, Burkina Faso, South Africa, and The Gambia with replication in 2868 African American adults. To evaluate the relationship between iron-associated genetic variants and infection risk, we examined the lead loci identified in the iron GWAS for association with severe malaria and bacteremia outcomes using the largest African case-control GWAS datasets (7957 malaria cases and 7746 controls; 1970 bacteremia cases and 4013 controls)^24,25^. We identify previously unreported African-specific genome-wide significant loci that are associated with iron homeostasis and risk of severe malaria and invasive bacterial infections.

## Results

### Characteristics of discovery sample

**Fig. 1:**
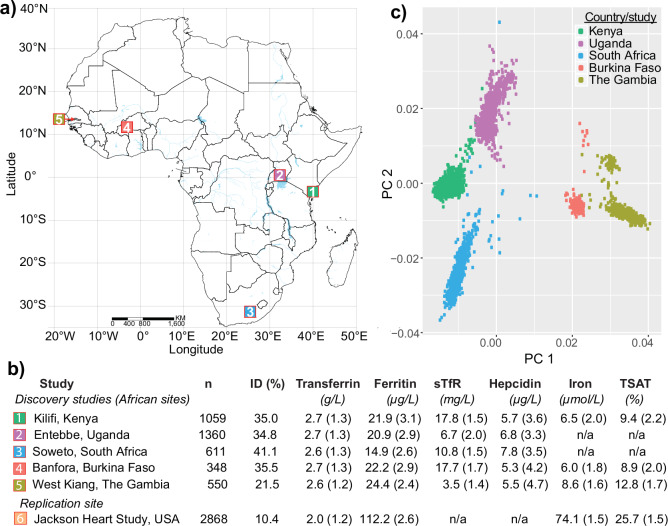
Characteristics of study populations. **a** Africa map showing the location of the discovery sites included in the current study. **b** Prevalence of iron deficiency (ID) and geometric means of iron markers by study site. ID was defined as serum ferritin <12 µg/L with no inflammation, or <30 µg/L in the presence of inflammation (C-reactive protein>5 mg/L or α1-antichymotrypsin >0.6 g/dL) in children <5 years or <15 µg/L with no inflammation or <70 µg/L in the presence of inflammation in children ≥5 years^70^. **c** Principal components plot of genotypes of the discovery study participants. *n* indicates biologically independent samples. sTfR soluble transferrin receptor, TSAT transferrin saturation, PC Principal Components, n/a not available.

To assess the genetic contribution to iron status in continental Africans, we performed a discovery GWAS analysis using data from five independent sites in Africa (Fig. 1a). A total of 3928 children (1059 Kenyan, 1360 Ugandan, 348 Burkinabe, 611 South African, and 550 Gambian), aged between birth and eight years were included. ID, inflammation, malaria parasitemia, stunting, and underweight were highly prevalent among study participants (Supplementary Table 1). Prevalence of ID and geometric mean concentrations of measured iron biomarkers (transferrin, ferritin, sTfR, hepcidin, serum iron, and TSAT) varied by study site (Fig. 1b). Principal component plots of the genotypes showed clustering of the study participants by geographical region and ethnolinguistic group (Fig. 1c). A summary of data quality control steps for each population is provided in Supplementary Tables 2 − 3.

### Discovery genetic association analyses

**Fig. 2:**
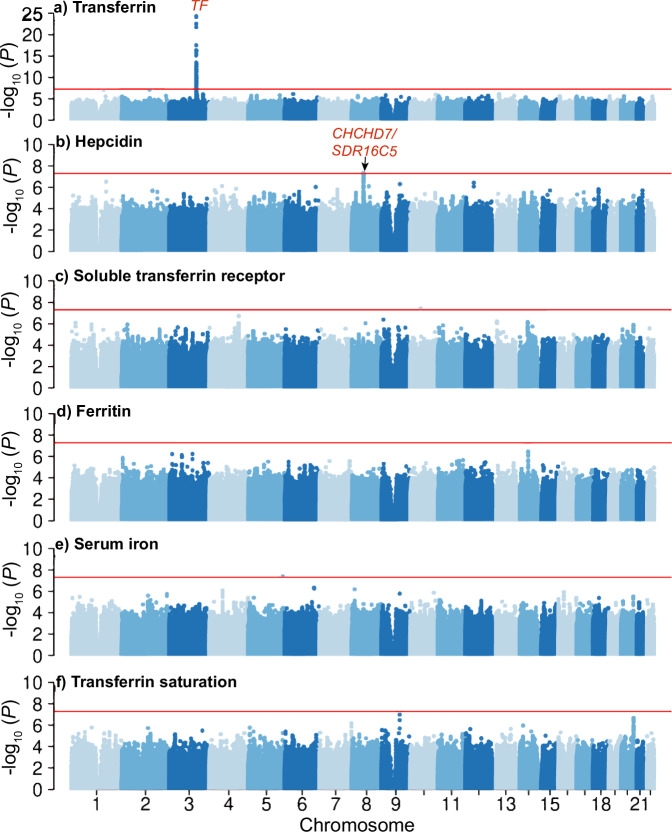
Manhattan plots of meta-analysis results of discovery samples. Manhattan plots for iron biomarkers including (**a**) Transferrin, (**b**) Hepcidin, (**c**) Soluble transferrin receptor, (**d**) Ferritin, (**e**) Serum iron, and (**f**) Transferrin saturation. Meta-analyses included all five African sites except for serum iron and transferrin saturation which were missing in Ugandan and South African samples. Genome-wide significant signals are annotated *TF*, for transferrin and *CHCHD7*/*SDR16C5* for hepcidin. Genetic variants were tested for association with iron biomarkers for each study site using linear mixed models and evidence was combined across sites using inverse-variance weighted meta-analysis in a fixed-effects framework. The red line indicates threshold for genome-wide significance (set at 5×10^−8^).

To identify variants associated with iron biomarkers, we performed individual GWAS within each of the African discovery sites (Supplementary Fig. 1) followed by meta-analysis. Figure 2 shows the distribution of association statistics for the GWAS of each of the six iron biomarkers after fixed-effects meta-analyses of all five African discovery sites. We applied a linear mixed model including a genetic relatedness matrix (GRM) to account for relatedness in the sample, and age and sex as covariates, and corrected any population stratification. There was minimal inflation of the association statistics (lambda ranged from 0.98 to 1.05, Supplementary Fig. 2). All lead SNPs were either genotyped or imputed with high quality (INFO > 0.95; Supplementary Data 1). Additional adjustment for inflammatory markers yielded similar results (Supplementary Table 4). Meta-analysis of GWAS results from all the African cohorts replicated a previously reported genome-wide significant locus for transferrin on chromosome 3 at the *TF* gene (Fig. 2a) and a previously unreported African-specific locus associated with hepcidin levels (Fig. 2b). We did not observe any loci at genome-wide significance (set at P < 5×10^−8^) after meta-analysis of GWAS results from all African discovery sites for sTfR, ferritin, serum iron, and TSAT (Fig. 2c–f).

### The *TF* locus

The *TF* locus is well characterized across ancestries. The lead SNP in our continental African transferrin GWAS was rs6762719 (P = 1.59×10^−27^) which is in strong LD (r^2^ = 0.9) with the lead common SNP (rs3811647) associated with transferrin levels in European ancestry transferrin GWAS^26^. The rs3811647 SNP (P = 3.45×10^−16^) was among the top SNPs in our African transferrin GWAS. After conditional analyses adjusting for rs6762719, we found another independent locus (lead SNP rs4854748) indicating at least two independent loci for transferrin (Table 1) as previously reported in African American populations^18^. This SNP (rs4854748) was in strong LD (r^2^ = 0.9) with the lead SNP, rs9872999, in the conditional analysis in African-Americans^18^. Notably, rs4854748 has been strongly associated with reduced total iron binding capacity (TIBC), a proxy for transferrin levels, in the largest European ancestry TIBC GWAS (P = 3.05×10^−578^)^15^. Genome-wide significant SNPs associated with transferrin levels in our discovery meta-analysis replicated in the Jackson Heart Study (JHS) and the largest European-ancestry TIBC GWAS^15^ (except for rs113656013 which is rare in Europeans; Supplementary Data 2), thus extending the transferability of known transferrin loci in Africa.

**Table 1:** Top independent SNPs associated with iron biomarkers in African children

SNP	Chr	Hg19 BP	EA	OA	Consequence	Gene	Source	EA Freq	Beta	SE	P
Transferrin (all African sites)*											
rs6762719	3	133480817	G	A	intronic	*TF*	Genotyped	0.25	0.28	0.03	1.59×10^−27^
rs4854748	3	133446881	T	C	intronic	*TF*	Imputed	0.20	−0.24	0.03	1.12×10^−16^
Transferrin (Uganda and Kenya)											
rs2905094	9	135902689	T	C	Upstream gene variant	*GTF3C5*	Imputed	0.16	−0.23	0.04	8.08×10^−9^
Hepcidin (all African sites)											
rs73596248	8	57134187	A	G	intergenic	*CHCHD7; SDR16C5*	Genotyped	0.06	0.27	0.05	4.51×10^−8^
sTfR (Kenya)											
rs552439837	4	144446525	G	A	intronic	*SMARCA5*	Genotyped	0.09	0.48	0.09	2.78×10^−8^

Chr, chromosome; Hg19 BP, human genome build 19 base pair; EA, effect allele; OA, other allele; EA Freq, effect allele frequency; Se, standard error; P, two-sided P value from an additive model.

*R^2^ between rs6762719 and rs4854748 was 0.03 in the African populations.

SNPs rs4854748 and rs2905094 were imputed with high accuracy (INFO > 0.95 across sites; see Supplementary Data 1).

### *GTF3C5*, an East African-specific locus for transferrin

**Fig. 3:**
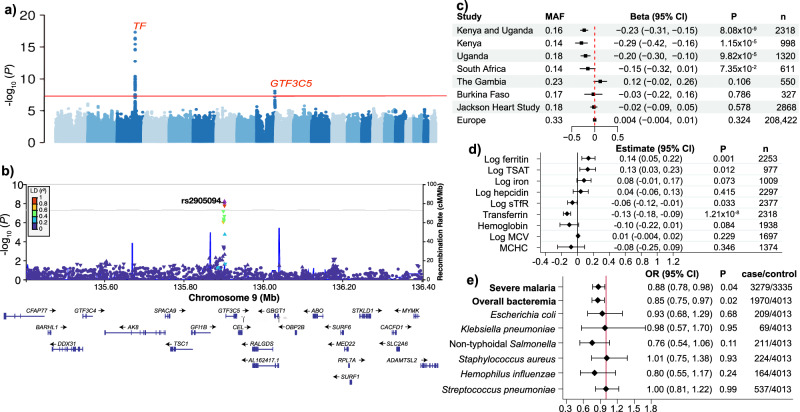
*GTF3C5* is a previously unreported locus associated with transferrin levels, severe malaria and bacteremia in East Africa. **a** Meta-analysis of transferrin GWAS results in East Africa (Kenya and Uganda). **b** A regional plot of the *GTF3C5* locus in East Africa. The most strongly associated variant, rs2905094 (P = 8.08×10^−9^), is represented by a purple diamond. Additional SNPs are colored by their correlation (r^2^) with rs2905094. Location and direction of transcription of protein-coding genes within the region are shown. **c** A forest plot by study site showing the minor allele frequency (MAF), GWAS beta (center) and 95% confidence interval (error bars), and additive model two-tailed GWAS p-values of the association between rs2905094 and transferrin levels. **d** Association between rs2905094 and iron biomarkers and red cell indices in Kenyan and Ugandan children. Estimates (center), 95% confidence intervals (error bars), and two-tailed p-values were derived from an additive linear regression model adjusted for age, sex, and study site. The association between rs2905094 and transferrin was tested at α = 0.05; associations with other iron biomarkers were considered exploratory and not adjusted for multiple comparisons. **e** A forest plot showing odds ratios (center), 95% confidence intervals (error bars), and two-tailed p-values of the association between rs2905094 and severe malaria, overall bacteremia, and common bacterial isolates. For severe malaria, we meta-analyzed East African (Kenya, Malawi and Tanzania) GWAS additive model estimates from the largest severe malaria GWAS^24^. For bacteremia, we performed additive logistic regression analyses adjusting for age, sex, and principal components. *n* indicates biologically independent samples.

Since genetic associations can be highly population-specific across Africa^27^, we performed meta-analysis by African region (i.e., East, West, and South Africa; Supplementary Fig. 3). For transferrin levels, we observed a previously unreported locus, *GTF3C5* (general transcription factor IIIC, polypeptide 5) on chromosome 9, with evidence in East Africa only (Kenya and Uganda; Fig. 3a). Three SNPs reached genome-wide significance (Supplementary Table 5). The lead SNP, rs2905094, was in high LD with the other two SNPs (Fig. 3b) and explained 1.42% of variation in transferrin levels. The T allele frequency of rs2905094 in East Africa was 16%, with allele frequencies of 14-23% across continental Africa and African American cohorts, and 33% in European populations (Fig. 3c). The rs2905094 SNP was associated with increased levels of ferritin and TSAT, decreased levels of sTfR (Fig. 3d), and a 19% reduction in ID (OR = 0.81 (95% CI 0.68, 0.97); P = 0.02) in East Africa.

We then used external reference data to annotate and evaluate the functional or regulatory impact of genome-wide significant variants and nearby gene(s). The three genome-wide significant SNPs (Supplementary Table 5) mapped to the *GTF3C5* (general transcription factor IIIC, polypeptide 5) gene. *GTF3C5* encodes transcription factor IIIC63 (TFIIIC63) which binds DNA to recruit transcription factor IIIB and RNA polymerase III to mediate the transcription of small noncoding RNAs, such as tRNAs. The lead SNP rs2905094 is located upstream of the transcription start site of *GTF3C5* and is a significant eQTL for *GTF3C5* (Beta = 0.07, SE = 0.03, *P* = 0.04, *n* = 593) in immortalized lymphoblastoid cell lines in the African Functional Genomics Resources (AFGR, https://github.com/smontgomlab/AFGR).

Given the above observations, we next performed haplotype analyses to investigate whether the rs2905094 variant might reside within distinct haplotypes in East Africa. We found two dominant haplotypes from chromosomes containing the rs2905094 mutant (T) allele (Supplementary Fig. 4). Haplotype 1 predominated in West Africa (The Gambia 62%, Burkina Faso 52%) but was less frequent in East and Southern Africa (Kenya 32%, Uganda 23%, South Africa 31%), whereas Haplotype 2 was absent in The Gambia, present at 9% in Burkina Faso and 15% in South Africa, and observed at moderate frequencies in East Africa (Kenya 33%, Uganda 20%; Supplementary Table 6). However, in haplotype-level analyses in East Africans, both Haplotypes 1 and 2 were associated with lower transferrin levels, and these effects disappeared after conditioning on rs2905094, indicating that the signal is driven by rs2905094 or an unidentified variant in strong LD with rs2905094 rather than by a haplotype-specific effect (Supplementary Table 7). Through fine-mapping, we identified a single credible set centred on rs2905094 which had the highest posterior inclusion probability (PIP = 0.57), with rs2905095 and rs2905089 contributing minor probabilities (Supplementary Fig. 5), consistent with a single causal signal at *GTF3C5*. Although rs2905094 is more common in Europeans, analysis of 1000 Genomes EUR data revealed two predominant haplotypes (Haplotype 3 and Haplotype 4; Supplementary Fig. 6) that could differ from African haplotypes, suggesting that the rs2905094-T allele may segregate on a different haplotype background in Europeans, potentially explaining the absence of an association in European iron GWAS.

Since *GTF3C5* knockdown has been shown to increase endocytosis and internalization of transferrin^28^, and cellular iron uptake influences risk of infection^29^, we investigated the association between rs2905094 and susceptibility to infection using large case-control GWAS of severe malaria^24^ and bacteremia^25^ (Supplementary Table 8). Since the effect of rs2905094 on transferrin levels was restricted to East Africa, we compared fixed effects meta-analyses of severe malaria GWAS data from East Africa (i.e. Kenya, Malawi, and Tanzania) and West Africa (The Gambia, Mali, Burkina Faso, Ghana, Nigeria, and Cameroon; Supplementary Fig. 7). We found that rs2905094 was associated with 12% (OR = 0.88, 95% CI 0.78, 0.98) protection against severe malaria in East Africa where it altered transferrin levels but was not associated with protection in West Africa where it did not alter transferrin levels (OR = 0.95; 95% CI 0.87, 1.03; Fig. 3e and Supplementary Fig. 7). rs2905094 was also associated with 15% (OR = 0.85, 95% CI 0.75, 0.97; P = 0.02) protection against bacteremia in Kenyan children (Fig. 3e).

### *CHCHD7/SDR16C5*, an African-specific locus for hepcidin

**Fig. 4:**
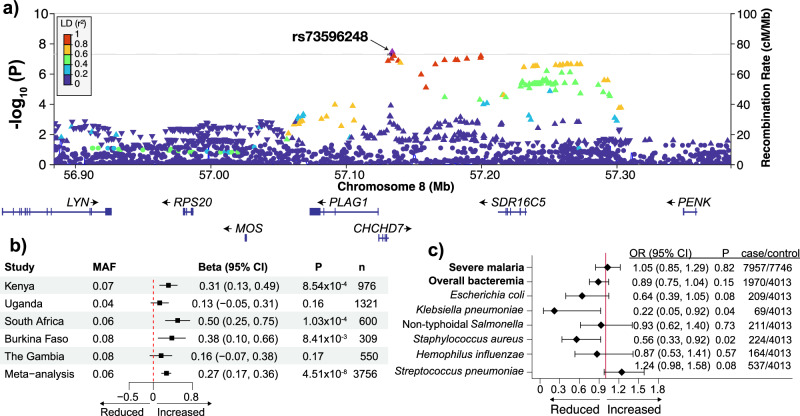
A previously unreported locus associated with hepcidin levels in African populations. **a** A regional plot for the locus. The most strongly associated variant, rs73596248 (P = 4.51×10^−8^), is represented by a purple diamond. Additional SNPs are colored by their correlation (r^2^) with rs73596248. Location and direction of transcription of protein-coding genes within the region are shown. **b** A forest plot by study site showing the minor allele frequency (MAF), beta (center) and 95% confidence interval (error bars), and additive model two-tailed GWAS p-values of the association between rs73596248 and hepcidin levels. Hepcidin data was not available for the Jackson Heart Study. **c** A forest plot showing the odds ratios (center) and 95% confidence interval (error bars) of the association between rs73596248 and severe malaria, overall bacteremia, and common bacterial isolates. For severe malaria, we meta-analyzed GWAS additive model estimates from African populations^24^. For bacteremia, we performed additive logistic regression analyses adjusting for age, sex, and principal components. *n* indicates biologically independent samples.

Meta-analysis of hepcidin GWAS results from all five African discovery sites yielded a previously unreported locus at genome-wide significance, found on chromosome 8 near the *CHCHD7* (Coiled-Coil-Helix-Coiled-Coil-Helix Domain Containing 7) and *SDR16C5* (Short Chain Dehydrogenase/Reductase Family 16 C Member 5) genes (Fig. 2b). The regional plot suggests that this association is within an extended haplotype (Fig. 4a) and we observed elevated normalized integrated haplotype score (iHS) values across all African cohorts (Supplementary Fig. 8 and Supplementary Data 3), indicative of extended haplotype homozygosity. The A allele of the lead SNP, rs73596248, occurred at a frequency of 6% in African populations with consistently increased hepcidin levels across all the African sites (Fig. 4b). rs73596248 explained 0.82% of variation in hepcidin levels. In contrast, the rs73596248 A allele was very rare in European and Latin American populations and was monomorphic in Asian populations (Supplementary Table 9). Associations between rs73596248 and other individual iron biomarkers and red cell indices did not reach statistical significance (Supplementary Fig. 9a).

The rs73596248 SNP lies downstream of *CHCHD7* and is an intergenic variant between the *CHCHD7* and *SDR16C5* genes. rs73596248 is a significant eQTL for *CHCHD7* (Beta = −0.60, SE = 0.16, *P* = 1.50×10^−4^, *n* = 593) in the AFGR immortalized lymphoblastoid cell lines. *CHCHD7* is a protein coding gene involved in mitochondrial function and is ubiquitously expressed but mainly in peripheral blood mononuclear cells and implicated in pleomorphic adenoma carcinoma and myoepithelial carcinoma through *CHCHD7-PLAG1* gene fusions that promote tumorigenesis^30^. The *SDR16C5* gene encodes short chain dehydrogenase reductase that oxidizes retinol to retinoic acid, which is known to suppress hepcidin production^31^. However, we did not find any rs73596248 eQTL data for *SDR16C5* in the eQTL databases.

Since hepcidin is implicated in the pathophysiology of malaria and bacterial infections in mouse models^12,13,32^, we determined the association between rs73596248 and these infections in the case-control GWAS of severe malaria and bacteremia described above^24,25^. We found that rs73596248 was associated with protection against bacteremia due to *Klebsiella pneumoniae* (OR = 0.22; 95% CI 0.05, 0.92; P = 0.04) and *Staphylococcus aureus* (OR = 0.56; 95% CI 0.33, 0.92; P = 0.02). Protection against *Escherichia coli* and overall bacteremia did not reach statistical significance, while severe malaria was not associated with the rs73596248 SNP (Fig. 4c).

### Dantu blood type is associated with sTfR levels in Kenya

**Fig. 5:**
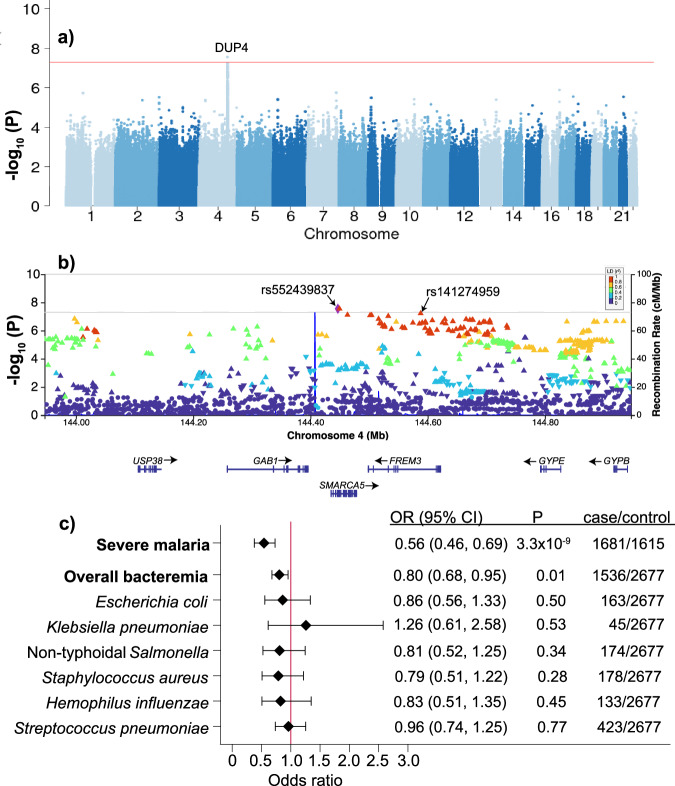
Genome-wide association study of soluble transferrin receptor in Kenya. **a** Manhattan plot of GWAS of soluble transferrin receptor (sTfR) in Kenya. **b** A regional plot showing correlation between the lead SNP, rs552439837 (purple diamond) and SNPs within the region. **c** A forest plot showing odds ratio (center), 95% confidence intervals (error bars), and two-tailed p-values of the association between rs141274959 and severe malaria, and between rs552439837 overall bacteremia, and common bacterial isolates. rs141274959 had an r^2^ = 0.9 with rs552439837 which was missing in the severe malaria GWAS. For severe malaria, we exponentiated GWAS additive model estimates from Kenya^24^. For bacteremia, we performed additive logistic regression analyses adjusting for age, sex, and principal components. *n* indicates biologically independent samples.

We observed a genome-wide significant locus at the *SMARCA5* gene associated with increased sTfR levels in Kenya (Fig. 5a). *SMARCA5* lies just outside a region of segmental duplication that contains the genes encoding the erythrocyte surface antigens glycophorin A and B. A complex structural variant at this locus, termed DUP4, encoding the Dantu NE blood group phenotype, has previously been shown to be under strong selective pressure due to its protective effect against malaria along the Kenyan coast^27,33^. The lead sTfR SNP in our GWAS, rs552439837, was in strong LD with DUP4 (e.g. r^2^ > 0.9 with the previously identified tag SNP, rs186873296)^33^, which was also associated with increased sTfR levels (P = 1.3×10^−6^). The alternate ‘G’ allele of rs552439837 occurred at a frequency of 9% in Kenya (Table 1) but as expected was rare outside Kenya (Supplementary Table 10). The regional plot suggests an extended haplotype underlying the association (Fig. 5b). The rs552439837 variant explained 3.8% of variation in sTfR levels and was associated with increased TSAT and serum iron concentrations (Supplementary Fig. 9b). Consistent with the East African-specific nature of the Dantu variant, we did not find any reported eQTL data for the top sTfR GWAS SNPs in available eQTL databases.

The Dantu blood group locus, marked by variants associated with soluble transferrin receptor (sTfR) levels, showed strong and concordant protective effects against both bacteremia and severe malaria. The lead sTfR GWAS SNP, rs552439837, was 20% protective against bacteremia (OR = 0.80; 95% CI 0.68, 0.95; *P* = 0.01; Fig. 5c), as previously shown using rs186873296^34^. Since rs552439837 was missing in the case-control study of severe malaria, we identified a tag SNP, rs141274959, from among the genome-wide significant sTfR GWAS SNPs (Supplementary Table 10). rs141274959 is in strong LD with rs552439837 (r^2^ = 0.9) and was highly protective against severe malaria (OR = 0.56; 95% CI 0.46, 0.69; *P* = 3.3×10^−9^). rs141274959 exhibited a 99.97% probability of colocalization between severe malaria and sTfR suggesting that this locus drives both traits.

### Transferability of established European iron GWAS SNPs

**Fig. 6:**
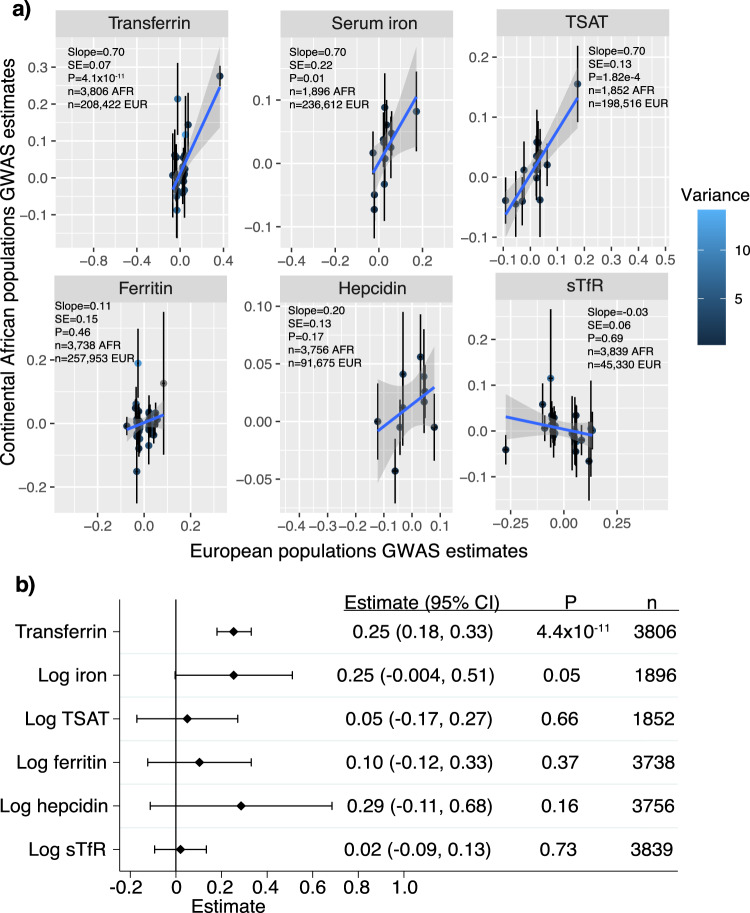
Replication of European ancestry iron GWAS in continental African populations. **a** Comparison of effect estimates for individual biomarkers of iron status between European and African ancestries. The slopes are inverse-variance weighted estimates. Estimates (center) reflect per-allele effect in standard deviation units of the inverse-normal transformed phenotype while error bars are 95% confidence intervals. Variance indicates SNP variance in European ancestry divided by SNP variance in continental African populations where SNP variance was calculated as 2 x estimate x (1 - effect allele frequency). **b** Transferability of polygenic risk scores (PRS) derived from European ancestry iron GWAS lead SNPs to continental African populations. For each iron biomarker, we calculated a PRS weighted using effect sizes reported in European ancestry iron biomarker GWAS and reflecting an increase in levels of the biomarker (included SNPs are shown in Supplementary Data 4 and 5). Estimates (center), 95% confidence intervals (error bars), and two-tailed p-values were derived from linear regression models of the association between iron biomarkers (measured in continental African populations) and European ancestry-derived PRS adjusting for age, sex, and study site. In continental African populations, we used transferrin as a proxy measure for total iron binding capacity (TIBC), which was reported in the European GWAS^15^. TIBC indicates the amount of iron that can be bound to transferrin. *n* indicates biologically independent samples. TSAT, transferrin saturation; sTfR, soluble transferrin receptor; AFR, continental African populations; EUR, European populations.

To investigate the transferability of European ancestry iron GWAS findings to African populations, we identified the estimates of European ancestry iron GWAS index SNPs^14,15^ in our meta-analyzed GWAS results of African children. Of the 138 index SNPs available in our GWAS, 69 (25 for ferritin, 20 transferrin, 9 serum iron, 6 hepcidin, 6 sTfR, and 4 TSAT) had the same direction of effect as the estimate in the European iron GWAS but only the *TF* locus was genome-wide significant (Supplementary Data 4 and 5). We observed some correlation in effect estimates for variants predicting transferrin and serum iron concentrations and TSAT between African and European populations, but little evidence of correlation for other iron markers (Fig. 6a). Additionally, most of the variant allele frequencies differed substantially between European and continental African populations. Notably, the *HFE* variants (rs1800562 and rs1799945) were very rare or monomorphic in African populations and the frequency of the effect allele (A) for the *TMPRSS6* rs855791 variant, was lower in African (7.7%) compared to European (43.1%) populations, although effect estimates were similar across the populations (Supplementary Data 4 and 5). We then calculated polygenic risk scores (PRS) for each iron biomarker using the European iron biomarkers GWAS index SNPs and similarly found that only the transferrin PRS replicated in African children (Fig. 6b).

## Discussion

Iron is a critical nutrient at the center of an ancient evolutionary arms race between the host and pathogen. This is particularly relevant in continental African populations with a historically high infectious disease burden, a high prevalence of iron deficiency and extensive genetic diversity. In this study, we report the first GWAS of iron status in continental African populations. We replicated two previously reported loci at the *TF* gene associated with transferrin levels^18,26^. We further discovered three African-specific genetic loci that influence iron status and risk of infection in African children. We identified a previously unreported East African specific locus, *GTF3C5*, implicated in intracellular iron uptake^28^, which was associated with reduced transferrin levels and protection against malaria and bacteremia. We also found a previously unreported locus within the *CHCHD7/SDR16C5* region, associated with increased hepcidin levels and protection against *K. pneumoniae* and *S. aureus* bacteremia. SNPs tagging the malaria protective DUP4/Dantu polymorphism^33^, were associated with increased sTfR levels in Kenyan children and protection against severe malaria and bacteremia. These region-specific findings underscore the existence of divergent patterns in LD among African populations^23^, and the evolutionary tension between maintaining sufficient iron for human physiology and restricting iron to limit pathogen proliferation. We further found limited transferability of findings from European iron GWAS to African populations, with PRS derived in European populations showing poor prediction.

Transferrin, synthesized in the liver, is the major iron-binding and transport protein that delivers iron to cells. It is highly conserved in humans and plays an important role in infection by sequestering iron away from pathogens, contributing to ‘nutritional immunity’^35^. Our transferrin GWAS analyses yielded three independent loci: two at the *TF* gene and one previously unreported locus at the *GTF3C5* gene. The two independent loci at the *TF* gene are well characterized across ancestries^15,26,36,37^. The *GTF3C5* locus was associated with reduced transferrin levels in East African populations. This observation aligns with findings that *GTF3C5* knockdown enhances transferrin endocytosis and internalization^28^, potentially explaining decreased plasma transferrin levels. Moreover, the lead SNP, rs2905094 was associated with elevated levels of serum ferritin, iron, and TSAT and a decrease in sTfR levels, in keeping with improved iron status. Indeed, we found that rs2905094 was associated with protection against ID suggesting a potential role in enhancing iron absorption.

Although the effect allele at the *GTF3C5* locus is common across African ancestry populations, and even more frequent in Europeans (33%), its association with transferrin levels was observed only in the East African cohorts. To investigate this geographic specificity, we examined local haplotype structure and found that rs2905094 occurs on multiple haplotypic backgrounds that differ in frequency across East and West Africa. Haplotype-level association analyses did not support a haplotype-specific effect suggesting that the observed association is not driven by a haplotype uniquely present in East Africa. Fine-mapping analyses identified rs2905094 as the variant with the highest posterior inclusion probability, although the possibility of a correlated variant in linkage disequilibrium cannot be excluded. Nonetheless, the population-specific nature of the association remains incompletely explained and may reflect differences in linkage disequilibrium, population structure, environmental context or gene-environment interactions, and unmodeled confounding, underscoring the importance of examining regional genomic diversity in African populations^20,23^.

We found that the *GTF3C5* locus was associated with reduced risk of severe malaria and bacteremia suggesting a link between iron metabolism and infection. We hypothesize two possible mechanisms. Firstly, an increase in intracellular iron within immune cells or improved iron status might play a role in improved immunity thereby protecting against infection^29,38^. Using a transgenic mouse model carrying a mutation in the transferrin receptor (thus limiting the ability of cells to internalize iron), Wideman et al reported impaired immune response and increased malaria parasitemia^29^. It is therefore possible that increased internalized iron improves immunity against pathogens. Secondly, upregulation of transferrin endocytosis^28^ may reduce circulating transferrin levels limiting the availability of iron, which is essential for the proliferation of *P. falciparum* and certain pathogenic bacteria^35,39^. Further studies are needed to test these hypotheses and elucidate the molecular pathways involved.

We identified a previously unreported African-specific locus between *CHCHD7* and *SDR16C5* associated with elevated hepcidin levels across the five African populations. SNPs within this locus occurred at a frequency of ~6% in African populations but are rare or monomorphic in non-African populations. Concordantly, we observed elevated normalized iHS values suggestive of recent positive selection acting on the derived allele at the locus probably to protect against pathogens. Hepcidin is the master regulator of systemic iron homeostasis and plays a crucial role in the body’s response to infection by inducing ‘nutritional immunity’^11,32^. The lead SNP, rs73596248, was associated with increased hepcidin levels and protection against *K. pneumoniae* and *S. aureus* bacteremia. In agreement, hepcidin has been shown to protect against *K. pneumoniae* and *S. aureus* infections by sequestering iron, limiting growth and improving survival in experimental models^32,40^. However, we found no association with severe malaria, consistent with our previous prospective study that found no relationship between hepcidin concentrations and malaria risk^9^. Together, these data support the view that this locus might represent an adaptive response to infectious disease pressure and raise important considerations for iron supplementation and fortification strategies.

We also found that mutations tagging the malaria-protective DUP4 haplotype on chromosome 4 were associated with increased sTfR levels and protection against severe malaria and bacteremia in Kenyan children. This allele is only found in Kenyan samples in our data, consistent with its global distribution which is rare outside East Africa. DUP4 is a structural mutation within the glycophorin gene region (comprising *GYPA*, *GYPB*, and *GYPE*) that encodes for the Dantu blood group antigen and is associated with protection against severe malaria in East Africa^33^. Recent analyses from the same population showed that another SNP tagging the Dantu polymorphism, rs186873296, and in strong LD with our lead SNP (r^2^ > 0.9), is associated with protection against bacteremia via its effect on malaria risk^34^. Malaria infection has previously been shown to strongly predispose individuals to bacteremia in malaria-endemic areas^41^. We further found a strong colocalization probability between severe malaria protection and increased sTfR levels, reflecting a shared genetic basis. While DUP4 reduces malaria risk, potentially lowering sTfR via reduced hemolysis^42^, it might paradoxically increase sTfR via altered red cell membrane dynamics affecting transferrin receptor cleavage or expression^43^. Although sTfR levels may not be directly linked to malaria infection in observational analyses^9^, elevated sTfR reflects expanded erythropoietic activity and can indicate ID, which is itself associated with protection against malaria infection^9^. This finding might also suggest that Dantu RBCs are younger than non-Dantu RBCs as previously shown^44^. However, the precise mechanisms linking erythropoiesis, iron metabolism, and malaria resistance remain to be fully elucidated, underscoring the complexity of host-pathogen-nutrient interactions.

We further found limited transferability of European iron GWAS findings to continental African populations. Using index variants identified in the largest iron GWAS in European populations^14,15^, we were only able to formally replicate prior reported loci in the *TF* locus, in African children. Notably, the *HFE* polymorphisms were rare or monomorphic in our GWAS, and it is well known that the hemochromatosis-causing variants are not observed in African populations. The *TMPRSS6* SNP, rs855791, although not as common in African compared to European populations, replicated in terms of effect size but not significance (P > 0.05) except for TSAT. Considering the low frequency of rs855791 in Africans, it is likely that we were underpowered to observe a statistically significant association. The *HFE* and *TMPRSS6* polymorphisms are often used in Mendelian randomization (MR) studies involving European populations^45,46^, but would have limited application in African MR and functional studies. All the other loci reported in European iron GWAS did not replicate (same direction of effect and P < 0.05) in our study populations. Similarly, only the transferrin PRS replicated in our African iron GWAS. However, our sample size was small compared to the large sample sizes reported in European GWAS. The European GWAS were also conducted in adults while the current study involved children, and regulation of iron homeostasis can differ across the life course. Different gene-environment interactions in Africa may further contribute to inconsistent replication results. Moreover, most variants had very different effect allele frequencies between African and European populations which might further explain the limited transferability and a need for African genomic studies.

The strengths of this study include a focus on diverse, understudied continental African ancestral groups and inclusion of a wide range of iron biomarkers. To our knowledge, this is the first GWAS of iron biomarkers within continental Africa. We identified previously unreported African-specific loci and assessed their impact on infection risk using the largest case-control studies of severe malaria and bacteremia. Potential limitations include heterogeneity in the availability of iron biomarker and red cell indices data across sites. For example, TSAT could not be calculated in Uganda and South Africa due to missing serum iron measurements, and ACT was used as a marker of inflammation instead of CRP in The Gambia. To minimize bias, all analyses were conducted within individual sites and combined by meta-analysis. Another limitation is that the bacteremia GWAS data were only available for Kenya, with limited sample sizes for specific bacterial isolates. Additionally, some lead SNPs were imputed, although imputation quality was high (INFO > 0.95), and variants with small effect sizes may not have been detectable with the current sample size. Larger genomic studies within Africa are needed to detect smaller-effect variants and refine genetic architecture across populations.

In conclusion, we identify African-specific genetic loci that influence iron biomarkers and risk of severe malaria and bacteremia. These findings have important implications for understanding genetic and environmental interactions shaping iron regulation and infection risk in African populations. While large European iron-GWAS have been foundational for several Mendelian randomization studies that clarified causal relationships relevant to clinical guidelines^45,46^, our results highlight the need for Africa-centric genomic data since genetic risk scores and instrumental variables identified in European populations were poorly predictive in African populations. Africa-specific data is needed to guide diagnostic screening and inform targeted interventions and equitable global health strategies. Our findings have potential public health relevance for iron supplementation programs since genetic differences in iron regulation may modify the balance between correcting deficiency and increasing infection risk, underscoring the importance of population-specific approaches. For instance, routine or universal iron supplementation or fortification, especially in the context of elevated hepcidin, may lead to iron accumulation in the gut or dysregulated iron distribution, potentially increasing susceptibility to enteric and systemic infections^8,47^. Future studies should explore the mechanistic pathways linking the identified variants to iron-pathogen interactions to inform safe and targeted interventions in African settings.

## Methods

### Ethical approvals

Individual study site ethical approvals were obtained. For Kilifi, Kenya (by the Scientific Ethics Review Unit of the Kenya Medical Research Institute (KEMRI/SERU/CGMR-C/046/3257/2983)); Entebbe, Uganda (locally by the Uganda Virus Research Institute (GC/127/12/07/32) and Uganda National Council for Science and Technology (MV625), and in the UK by the London School of Hygiene and Tropical Medicine (A340) and Oxford Tropical Research (OTR) (39-12, 42-14 and 37-15) Ethics Committees); Banfora, Burkina Faso (by Ministere de la Recherche Scientifique et de l’Innovation in Burkina Faso (2014-12-151) and the OTR Ethics Committees (41-12)); Soweto, South Africa (by the University of Witwatersrand Human Research (M130714) and the OTR Ethics Committees (1042-13 and 42-14)); and West Kiang, The Gambia (by the Gambian Government / Medical Research Council Ethics Committee (874/830)). The Jackson Heart Study (JHS) was approved by the IRB of the University of Mississippi Medical Center, Jackson State University, and Tougaloo College. Informed written consent was obtained from all children’s parents or guardians.

### Study populations

Our discovery studies included five community-based cohorts of children from sub-Saharan Africa in Kenya, Uganda, Burkina Faso, South Africa, and The Gambia. Our replication population included African American adults from the Jackson Heart Study (JHS) in Mississippi, USA. We further analyzed GWAS data on severe malaria and bacteremia to assess whether the identified iron-related SNPs were associated with susceptibility to these infections. Below is a description of each of the study sites.

#### Discovery cohorts

##### Kilifi, Kenya

This included an ongoing rolling cohort evaluating malaria immunity in children in Kilifi, along the coast of Kenya^48^. Within this cohort, children were followed-up for malaria episodes up to 8 years of age with weekly follow-ups and annual single cross-sectional surveys during which anthropometry measurements, auxiliary temperature, and blood samples were taken. Iron and inflammatory markers were measured from blood samples collected at annual cross-sectional surveys (between 1998 and 2016) based on the availability of plasma samples archived at −80 °C.

##### Entebbe, Uganda

The *Entebbe Mother and Baby Study (EMaBS)* is a prospective birth cohort that was originally designed as a randomized controlled trial to test whether anthelminthic treatment during pregnancy and early childhood was associated with differential response to vaccination or incidence of infections including malaria (http://emabs.lshtm.ac.uk/)^49^. Pregnant women from Entebbe in their second or third trimester of pregnancy were initially enrolled at Entebbe Hospital antenatal clinic. Livebirths from these mothers were enrolled into the EMaBS study. Blood samples were collected in vacutainer tubes containing ethylenediaminetetraacetic acid (EDTA) at birth and at subsequent birthdays up to five years of age. Iron and inflammatory biomarkers were measured from a single annual visit based on the availability of stored samples.

##### Banfora, Burkina Faso

The *VAC050 ME-TRAP Malaria Vaccine Trial* recruited infants between the ages of 6 and 18 months living in the Banfora region of Burkina Faso to participate in a Phase 1/2b clinical trial of the safety, immunogenicity and efficacy of a viral-vectored prime-boost liver-stage malaria vaccine^50^. Samples from a total of 350 infants were then selected based on suitability of samples for DNA extraction. Plasma samples were available from the infants at multiple time-points following receipt of the experimental vaccine. Samples from individuals taken at time points as close to the 12-month age as possible were prioritized for this study.

##### South Africa

*The Soweto Vaccine Response Study* consisted of infants born in Chris Hani Baragwanath Hospital living in the Soweto region of Johannesburg, South Africa who were recruited from vaccine trials^51^ that are coordinated by the Respiratory and Meningeal Pathogens Unit (http://www.rmpru.com/). Mothers of the infants were approached if their infants had received all Expanded Programme on Immunization vaccines up to six months of age. The infants were sampled prospectively at six months of age and at 12 months after receipt of measles vaccine at nine months. Single whole blood samples were collected in EDTA vacutainer tubes for measurement of iron and inflammatory markers and DNA extraction.

##### The Gambia

*West Kiang study:* All children aged two to six years (except those with chronic illness) were recruited from 10 rural villages in the West Kiang region of The Gambia during the start of a malaria season to initially investigate seasonal genetic effects of iron status, hemoglobin and haptoglobin concentrations between July and August 2001^52^. Ethnic groups were Mandinka (nine villages) and Fulani (one village). All children had a clinical examination, anthropometric measurements, and a three-day course of mebendazole for potential hookworm infection. A blood sample was collected for measurement of iron and inflammatory markers and DNA extraction.

#### Replication cohort

##### Jackson Heart Study

This is a population-based longitudinal study of African American adults aged ≥21 years living in the Jackson, Mississippi metropolitan area in the USA^53^. This study was designed to evaluate risks of cardiovascular disease^53,54^. Iron and inflammatory biomarkers were measured from blood samples collected at a single baseline clinic visit. Whole blood was used to extract DNA using Puregene reagents (Gentra System, Minneapolis, USA).

#### Infection GWAS

##### MalariaGEN consortium

This was a GWAS study of severe malaria in ~17,000 severe malaria cases and population controls from 11 countries in Africa (Kenya, Malawi, Tanzania, The Gambia, Mali, Burkina Faso, Ghana, Nigeria, Cameroon), Asia (Vietnam), and Oceania (Papua New Guinea)^24^. Sample collection, processing, genotyping, quality control, imputation, GWAS, and population characteristics are described in detail elsewhere^24,55^. Severe malaria was defined according to WHO criteria^56^ and included diagnoses of severe malaria anemia, cerebral malaria, and other malaria-related symptoms. Association testing for each variant and severe malaria was performed using SNPTEST software in each study population adjusting for principal components under additive, dominant, recessive, heterozygote and general models of association. In the current study, we extracted additive model estimates for African populations. For the East-Africa specific *GTF3C5* lead SNP, rs2905094, we retrieved population-specific results from all of the nine African populations in the MalariaGEN database and applied fixed-effects meta-analyses by East (Kenya, Malawi, and Tanzania) and West (The Gambia, Mali, Burkina Faso, Ghana, Nigeria, and Cameroon) African sites. For the association between the hepcidin lead SNP, rs73596248 and severe malaria, we meta-analyzed estimates from all the nine African populations in the MalariaGEN database. Since the sTfR GWAS signal is specific to Kenyan populations, we used data from the MalariaGEN Kenyan GWAS to determine the association between the top sTfR GWAS SNPs and severe malaria. The lead SNP, rs552439837, was not present in the severe malaria case–control dataset; therefore, we used estimates for rs141274959, a genome-wide significant sTfR GWAS variant in strong LD with rs552439837 (r² = 0.9).

##### The Kenyan Bacteremia GWAS

This GWAS included 1970 Kenyan children (<13 years old) admitted to Kilifi County Hospital between 1998 and 2010 with culture-confirmed community-acquired bacteremia, and 4013 healthy community controls^25^. Controls were infants aged 3-12 months recruited between 2006 and 2008 from the same geographic area through the Kilifi Genetic Cohort Study. All participants were residents of Kilifi County on the coast of Kenya. Genotyping was performed in two phases: the discovery phase employed the Affymetrix SNP 6.0 array (787,861 SNPs) in 1536 cases and 2677 controls, while the replication phase used the Illumina ImmunoChip (143,100 SNPs) in an additional 434 cases and 1336 controls, yielding a total of 1970 bacteremia cases and 4013 controls. Whole-genome imputation was carried out using SHAPEIT and IMPUTE2 with the 1000 Genomes Phase 3 reference panel. Demographic data for cases and controls and distribution of the most common bacterial species in the discovery and replication sets are described elsewhere^25^. In the current study, associations between conditionally independent iron-related SNPs and bacteremia were assessed using additive logistic regression models, adjusted for age, sex, and principal components. Analyses were performed for overall bacteremia and then stratified by the most common bacterial species.

### Assays for iron and inflammatory markers

The assayed biomarkers of iron (ferritin, soluble transferrin receptors (sTfR), hepcidin, serum iron, transferrin, and unsaturated iron binding capacity (UIBC) and inflammation (C-reactive protein (CRP) and α_1_-antichymotrypsin (ACT) are shown in Supplementary Table 11. Since iron biomarkers can be affected by inflammation or infection^1^, it is important to account for inflammation when defining iron deficiency. We defined inflammation as CRP > 5 mg/L or ACT > 0.6 g/L (in Gambian children), consistent with established guidelines for identifying inflammation in children^57^. The Gambian hepcidin values were harmonized with the rest of the cohorts’ hepcidin values (measured using high sensitive DRG kit) by converting to the old DRG hepcidin assay values and then to the new high sensitive DRG hepcidin assay values^58^. Transferrin saturation (TSAT) was calculated as (serum iron (µmol/L)/transferrin (g/L) x 25.1) x 100 or as (serum iron/ UIBC + serum iron) x 100^59^. Since EDTA chelates iron, serum iron measurements (and TSAT) were missing in Uganda and South Africa where blood samples were collected in EDTA vacutainer tubes. Transferrin (g/L) in JHS was calculated as 0.7 x total iron binding capacity (TIBC) [µg/dL] x 0.01. Transferrin in The Gambia was similarly calculated but after calculating TIBC as UIBC + serum iron. Red cell indices data included hemoglobin levels, Mean Corpuscular Volume (MCV), and Mean Corpuscular Hemoglobin Concentration (MCHC), measured using automated blood analyzers (Beckman Coulter).

### Genotyping and imputation

Genotyping for DNA extracted from Uganda, Burkina Faso and South Africa was performed using the Illumina HumanOmni 2.5M-8 (‘octo’) BeadChip array version 1.1 (Illumina Inc., San Diego, USA), at the Genotyping Core facilities at the Wellcome Trust Sanger Institute. Genotyping for Kenyan and Gambian samples was performed using H3Africa Custom Genotyping Array v1.0 at the Wellcome Centre for Human Genetics, University of Oxford. JHS samples were genotyped using the Affymetrix Genome-Wide Human SNP Array 6.0. Genotypes were called from intensities using Illuminus and GenCall clustering algorithms in GenomeStudio (Illumina Inc., San Diego, USA) incorporating data from pre-determined genotypes.

We performed quality control (QC) for samples and single nucleotide polymorphisms (SNPs) using SNPs mapped to Human Genome Build 37. Using the appropriate strand files (http://www.well.ox.ac.uk/~wrayner/strand/), we removed low quality variants (those that mapped to multiple regions within the human genome or did not map to any region) and duplicate SNPs. Only autosomal SNPs were included in downstream QC analyses. We used a standardized H3A GWAS pipeline (https://github.com/h3abionet/h3agwas) to perform sample and SNP QC separately for each cohort (using identical steps)^60,61^. Samples having discordant sex information, call rates <98%, relatedness cutoffs of between 11 and 70% (accounts for cryptic relatedness), and heterozygosity <0.15 or >0.343 were removed. SNPs were retained if they passed thresholds of >99% call rate, minor allele frequency (MAF) > 0.01, and Hardy-Weinberg equilibrium (HWE) adjusted p-value > 0.008.

We imputed our quality-controlled genotypes using the African Genome Resources haplotype reference panel that contains 4,956 samples from 1000 Genomes Phase 3 and ~2000 Ugandan samples and ~100 Ethiopians/Egyptians/Namibians/South Africans (n = 93,421,145 biallelic SNPs). This reference panel is hosted by the Wellcome Sanger Institute (https://imputation.sanger.ac.uk/)^62^. We filtered out the resulting imputation dataset for SNPs with information score ≥ 0.3 and MAF ≥ 0.01 for association analyses.

### Statistical analyses

We used inverse-normal transformation to normalize the distribution of iron biomarkers in R version 4.2.3. We performed association testing for each study site using genome-wide complex trait analysis (GCTA) software version 1.24.4^63^ fitting a linear mixed model including a genetic relatedness matrix (GRM) as a random effect. We included age and sex as covariates. We also assessed the impact of additional adjustment for inflammatory markers. Inverse normal transformation measures were used throughout our GWAS analyses, and all results are reported as such. We meta-analyzed our discovery study-specific summary statistics using METASOFT version 2.0.1 that reports both fixed and random effects^64^. Results were visualized using Manhattan and QQ plots generated using R packages *GenABEL* and *qqman*. Inflation factors (*lambda*) were calculated to check for population stratification. We used LocusZoom.js v0.12.0 (https://my.locuszoom.org/) to create GWAS regions and added LDs calculated using Plink version 1.9 from our discovery cohorts data. The R package *coloc* was used to estimate the probability of colocalization using the coloc’s default prior probability of colocalization of 1×10^−5^ and considered a posterior colocalization probability > 80% as a threshold for evidence of colocalization.

Genome-wide significant SNPs (P < 5×10^−8^) from discovery GWAS meta-analyses were selected for testing in the JHS replication study. We considered a GWAS result to replicate if the effect in the replication was in the same direction as in the discovery sample, and if the association in the replication sample was statistically significant (P < 0.05).

To evaluate differences in the genetics of iron between European and African ancestry populations, we analyzed transferability of iron GWAS findings between continental African and European populations. We identified index SNPs from the largest genome-wide meta-analysis of European ancestry iron biomarkers GWAS^14,15^ for replication in our African iron GWAS. All lead/independent SNPs reported in the European ancestry iron GWAS were identified in African children with available iron GWAS data. We then compared effect sizes and P values between European and African populations. Plots comparing the effect estimates were generated using the R package *ggplot2*. A SNP was considered to replicate if the effect in Africans was in the same direction as in the European iron GWAS, and if the association in Africans was statistically significant (P < 0.05). For each iron biomarker, we further calculated a PRS^65^, weighted using effect sizes reported in European GWAS and reflecting an increase in levels of the biomarker. A linear regression model adjusted for age, sex and study site was used to determine any association between the PRS and iron biomarkers measured in African children. In Africans, we used transferrin as a proxy measure for total iron binding capacity (TIBC), which was reported in European iron GWAS. TIBC indicates the amount of iron that can be bound to transferrin.

Since iron status might influence risk of malaria and bacterial infections^39,66^, we investigated the association between our identified African-specific SNPs and these infections using the largest case-control GWAS of severe malaria^24^ and bacteremia^25^ as described above. Since the *GTF3C5* lead SNP, rs2905094, showed an East-African specific effect on transferrin levels, we meta-analyzed severe malaria GWAS results from East African countries only (Kenya, Malawi and Tanzania). Since the *SDR16C5* lead SNP appeared across all five iron GWAS African sites, we meta-analyzed severe malaria results from all nine MalariaGen African sites (The Gambia, Mali, Burkina Faso, Ghana, Nigeria, Cameroon, Malawi, Tanzania, and Kenya) using the ‘*meta’* R package assuming fixed effects. For the DUP4 locus lead SNP, which is Kenyan specific and very rare in other populations across our cohorts or in reference panels such as 1000 G, we extracted its effect size in the Kenya severe malaria GWAS only. For SNP-bacteremia associations, we identified our African-specific SNPs in the largest GWAS of bacteremia in African populations^25^, merged the genotypes with the hospital clinical data, and conducted logistic regression analyses adjusting for age, sex, and principal components.

### Functional annotation and statistical fine-mapping

To identify independent loci, we performed statistical fine-mapping by conducting conditional forward stepwise regression. This entailed conditioning on the lead SNP from our GWAS by treating it as an adjusting covariate and testing the remaining SNPs. Once a new independent secondary signal was identified, we performed sequential testing by adjusting for the lead SNP until no conditional tests were statistically significant.

To identify variants driving the East African *GTF3C5* locus, we performed statistical fine-mapping using the *susieR* package^67^ in R version 4.2.3 based on meta-analyzed transferrin GWAS summary statistics from East African cohorts (Kenya and Uganda). A region-specific LD matrix, derived from the merged East African genotype data, was used to accurately model local LD structure. We specified up to 10 single-effect components (L = 10) to allow for multiple independent causal signals within the locus. Posterior inclusion probabilities (PIPs) were estimated for each variant, and 95% credible sets were defined to identify variants most likely to drive the association signal.

We performed functional annotation of lead GWAS variants using the Ensembl Variant Effect Predictor (VEP, GRCh37, https://www.ensembl.org/Tools/VEP), reporting the most severe consequence per variant based on canonical transcripts. To identify candidate target genes, we used the FUMA GWAS platform (https://fuma.ctglab.nl/), applying three complementary mapping strategies: (1) positional mapping within ±10 kb of gene boundaries, (2) cis-eQTL mapping using data from GTEx v8 across multiple tissues, and (3) chromatin interaction mapping based on Hi-C datasets from relevant cell types. In parallel, we queried the African Functional Genomics Resource (https://github.com/smontgomlab/AFGR) to identify eQTLs in African ancestry populations. Genes implicated by any of these approaches were considered candidate target genes.

### Haplotype analyses

Haplotype patterns were derived from the imputed and phased genotypes using a custom R script that computed Manhattan distances followed by hierarchical clustering using the *hclust* function in R software (version 4.0.2). The resulting clusters were visualized with heat maps, and haplotype patterns were identified by visually assessing regions of homogeneity. We focused on a 72 kb region defined by 209 SNPs surrounding the rs2905094 SNP. Haplotype pattern analysis was performed separately for the ancestral and derived alleles of rs2905094, both across all countries combined and for each country individually. Since rs2905094 is common in European ancestry populations, we also analyzed the *GTF3C5* haplotype configuration using 1000 Genomes Project EUR data.

To determine whether the association signal at *GTF3C5* was driven by a specific haplotype carrying the derived rs2905094-T allele, we performed haplotype-level association analyses for transferrin levels among East African participants. Haplotype dosages (0/1/2) were estimated from phased genotype data and incorporated into linear regression models of inverse-normalized transferrin levels, adjusting for age, sex, study site, and the first ten principal components of ancestry.

### Selection scan using integrated haplotype scores

Since the LD pattern around the *CHCHD7/SDR16C5* locus on chromosome 8 was suggestive of an extended haplotype, we investigated extended haplotype homozygosity (EHH) patterns for each of the five African sites. Variants were restricted to chromosome 8 positions 57,050,000–57,300,000 bp (GRCh37/hg19 coordinates) to encompass the LD block surrounding the lead hepcidin GWAS variant (rs73596248). SNP positions within the target region were used to interpolate recombination distances from the 1000 Genomes Project Phase 3 genetic map. Integrated haplotype scores (iHS) were computed using selscan v2.1.0^68^. SNPs with MAF ≥ 0.01 were included. The iHS statistic was calculated for variants in the target region, measuring the decay of EHH for ancestral and derived alleles. Using selscan’s normalization procedure, raw iHS were standardized by allele frequency bins (n = 10) across the genome to account for variation in allele frequencies. Haplotype signals were visualized using *ggplot2* package in R v4.3.2, with loess smoothing applied to highlight local trends in normalized iHS values across the genomic region.

### GWAS sample size and power

Power for the GWAS studies was calculated using the formulae given in Visscher et al 2017^69^, implemented in: https://github.com/kaustubhad/gwas-power. Given an allele frequency ≥ 5% and effect size ≥ 0.33 standard deviation (SD), the overall discovery sample size of 3928 provided more than 80% power to observe true genotypic and phenotypic associations at a genome-wide significance level (type I error=5×10^−8^). For allele frequency ≥10%, we had over 80% power to see effect sizes ≥ 0.24 SD. For the association between lead SNPs and severe malaria or bacteraemia, we estimated that the severe malaria dataset (7957 cases, 7746 controls) had ~80% power to detect an odds ratio ≥1.15 for a variant with MAF = 5% at α = 0.05, while the bacteremia dataset (1970 cases, 4013 controls) had ~80% power to detect an odds ratio ≥1.40.

### Reporting summary

Further information on research design is available in the Nature Portfolio Reporting Summary linked to this article.

## Supplementary information

Supplementary Information

Reporting summary

Transparent Peer Review file

## Source data

Source Data 1

Source Data 2

Source Data 3

Source Data 4

Source Data 5

## Data Availability

Summary statistics for the genome-wide association tests of imputed data for iron biomarkers in continental African populations reported in this study have been deposited in the Harvard Dataverse at 10.7910/DVN/ZGGN2F. The supplementary data generated in this study are provided in the Supplementary Information and Supplementary Data files. The European GWAS meta-analysis summary level data for serum ferritin, serum iron, total iron-binding capacity, and transferrin saturation used in this study are available from NTNU Open Research Data (10.18710/S9TJEL), while those for hepcidin and soluble transferrin receptors were obtained from the NHGRI-EBI GWAS Catalogue (hepcidin: accession number GCST90451683; soluble transferrin receptor: accession number GCST90451684). Source data are provided with this paper.

## Code availability

All analyses used preassigned code defined within software packages that are publicly available as described in Methods. Any other requests for clarifications may be sought from the corresponding authors.
