# Supplementary material for: African-specific genetic loci determine iron status and risk of severe malaria and bacteremia in African children
Source: Nat Commun. 2026 Apr 7;17:7068. doi: 10.1038/s41467-026-71567-w (PMC13392482; doi:10.1038/s41467-026-71567-w)
Supplement: Supplementary file 1 — Supplementary Information [file 41467_2026_71567_MOESM1_ESM.pdf]

Supplementary Information for

African-specific genetic loci determine iron status and risk of severe malaria and bacteremia in African children

John Muthii Muriuki[1*], Alexander J Mentzer[2,3], Gavin Band[2], Amanda Y Chong[2], Alex W Macharia[1], Reagan M Mogire[1,4], Kelvin Mokaya Abuga[1], Ruth Mitchell[5], James J Gilchrist[6], Emily L Webb[7], Francis M Ndungu[1], Laura M Raffield[8], Lynette Ekunwe[9], Amy R Bentley[4], Sodiomon B Sirima[10], Shabir A Madhi[11], Adrian V S Hill[2,12], Andrew M Prentice[13], Philip Bejon[1,14], Gibran Hemani[5], George Davey Smith[5], Manjinder S Sandhu[15], Alison M Elliott[16,17], Thomas N Williams[1,18,19], Adebowale Adeyemo[4], and Sarah H Atkinson[1,18,20*]

[*]Corresponding authors. Email: jmuriuki@kemri-wellcome.org; satkinson@kemri-wellcome.org

The PDF file includes:

Supplementary Figures

Supplementary Figure 1: Manhattan plots for each African study site.
Supplementary Figure 2: QQ plot for the discovery GWAS of iron biomarkers.
Supplementary Figure 3. Meta-analysis of transferrin GWAS results by African region.
Supplementary Figure 4: Haplotype structures, linkage disequilibrium, and genetic map of the *GTF3C5* gene region.
Supplementary Figure 5: Fine-mapping of *GTF3C5* locus in East African populations.
Supplementary Figure 6: Haplotype structure of the *GTF3C5* gene region in European 1000G data.
Supplementary Figure 7: Meta-analyses of the effect sizes of the *GTF3C5* lead SNP, rs2905094 and severe malaria.
Supplementary Figure 8: Normalized integrated haplotype score (iHS) across chr8:57.05 - 57.30 Mb within *CHCHD7/SDR16C5* locus.
Supplementary Figure 9: Association between a) hepcidin and b) soluble transferrin receptor GWAS lead SNPs and iron biomarkers.

Supplementary Tables

Supplementary Table 1. Characteristics of study participants
Supplementary Table 2. Summary of quality control (QC) steps for each population.
Supplementary Table 3. Number of variants imputed into individual datasets.
Supplementary Table 4. Comparison of results adjusted for age and sex and those additionally adjusted for inflammatory markers for top independent SNPs associated with iron biomarkers in African children
Supplementary Table 5. Genome-wide significant SNPs associated with transferrin levels at *GTF3C5* locus in meta-analysis of Kenya and Uganda and how they compare with other populations
Supplementary Table 6. Distribution of haplotypes carrying the rs2905094-T allele at the *GTF3C5* locus across African populations
Supplementary Table 7. *GTF3C5* haplotype-level association analysis in East Africa.
Supplementary Table 8. Summary statistics for bacteremia and severe malaria GWAS at lead African-specific iron-related SNPs
Supplementary Table 9. Top SNPs associated with hepcidin levels in continental African populations and their frequencies in European, Asian and Latin American populations
Supplementary Table 10. Top SNPs associated with soluble transferrin receptor levels in Kenya and how they compare with other continental African populations
Supplementary Table 11. Laboratory assays for iron and inflammatory biomarkers by site

Supplementary References

Supplementary Figures

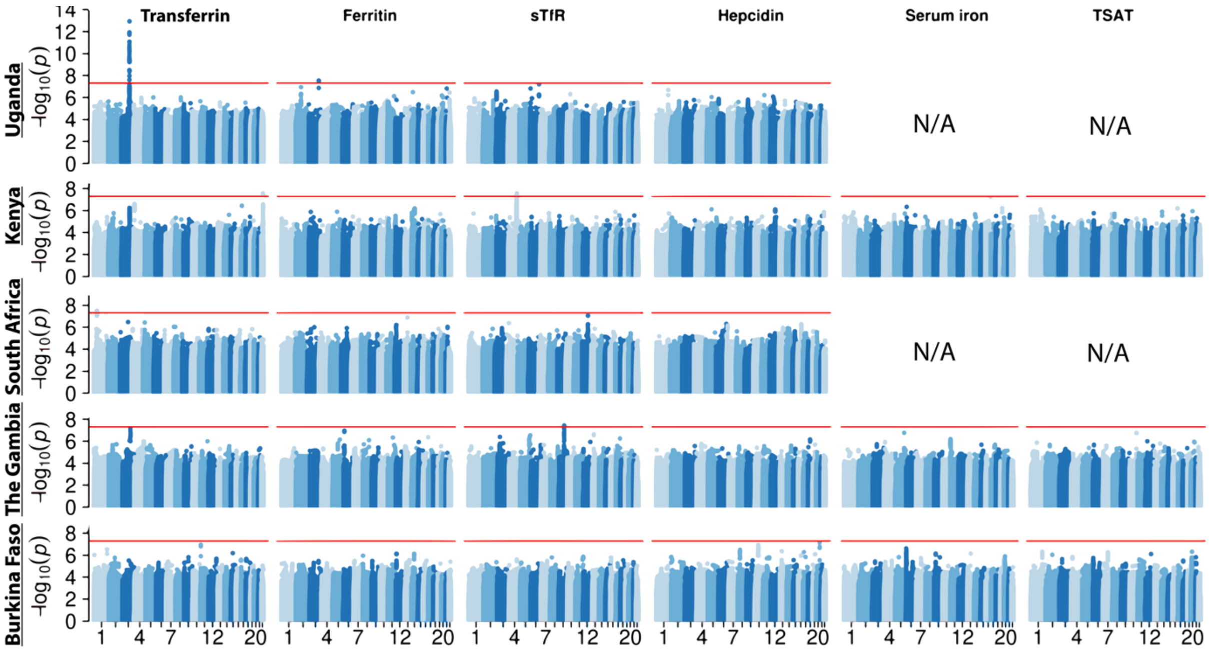

Supplementary Figure 1: Manhattan plots for each African study site. Several genome-wide significant associations observed in individual country-level analyses (for example, sTfR in The Gambia) were driven by rare variants (MAF < 2%). Due to their low frequency and limited power for replication, these signals were not explored further in the current study. N/A indicates data not available.

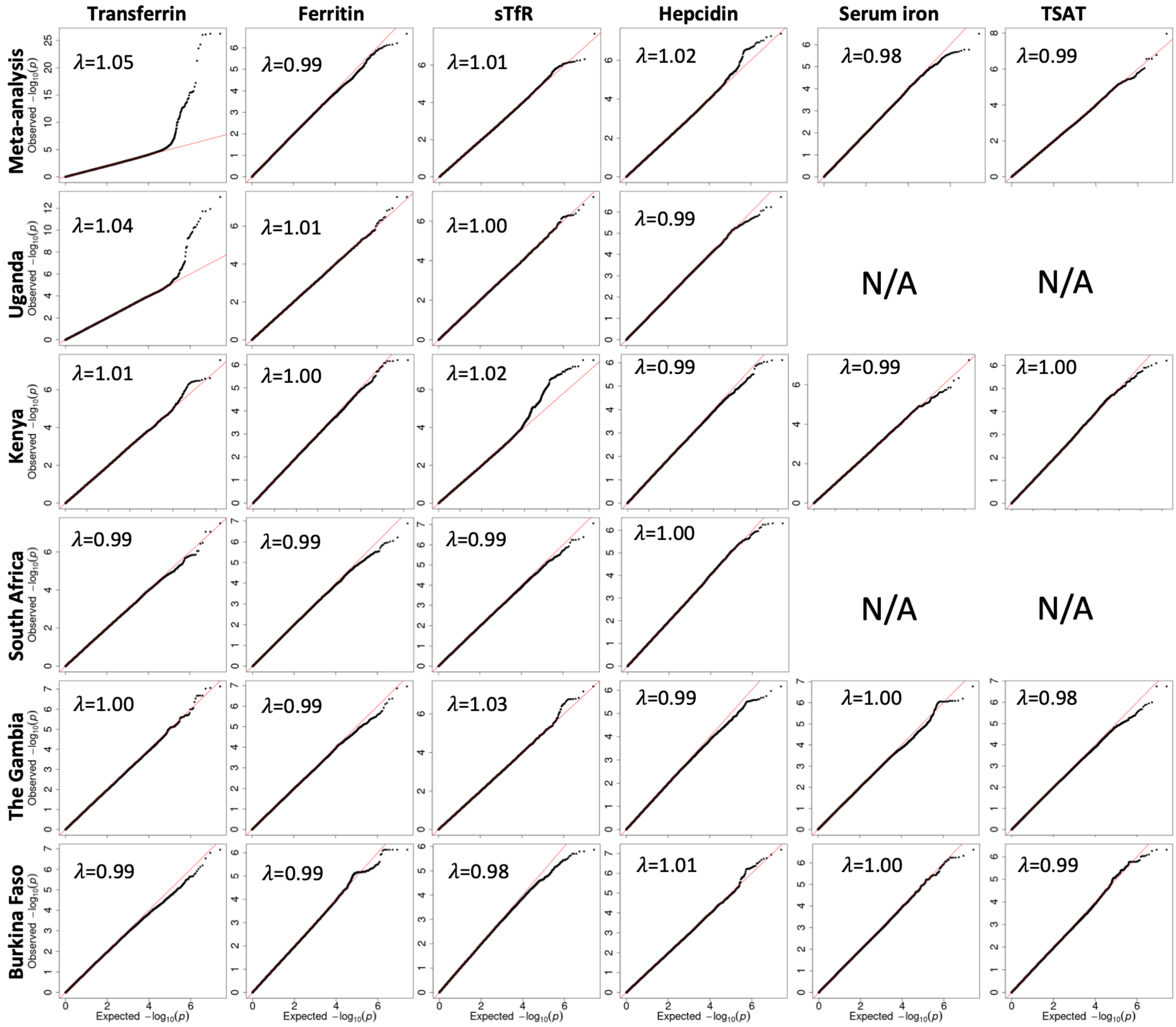

Supplementary Figure 2: QQ plot for the discovery GWAS of iron biomarkers. λ indicates inflation factor. N/A indicates data not available.

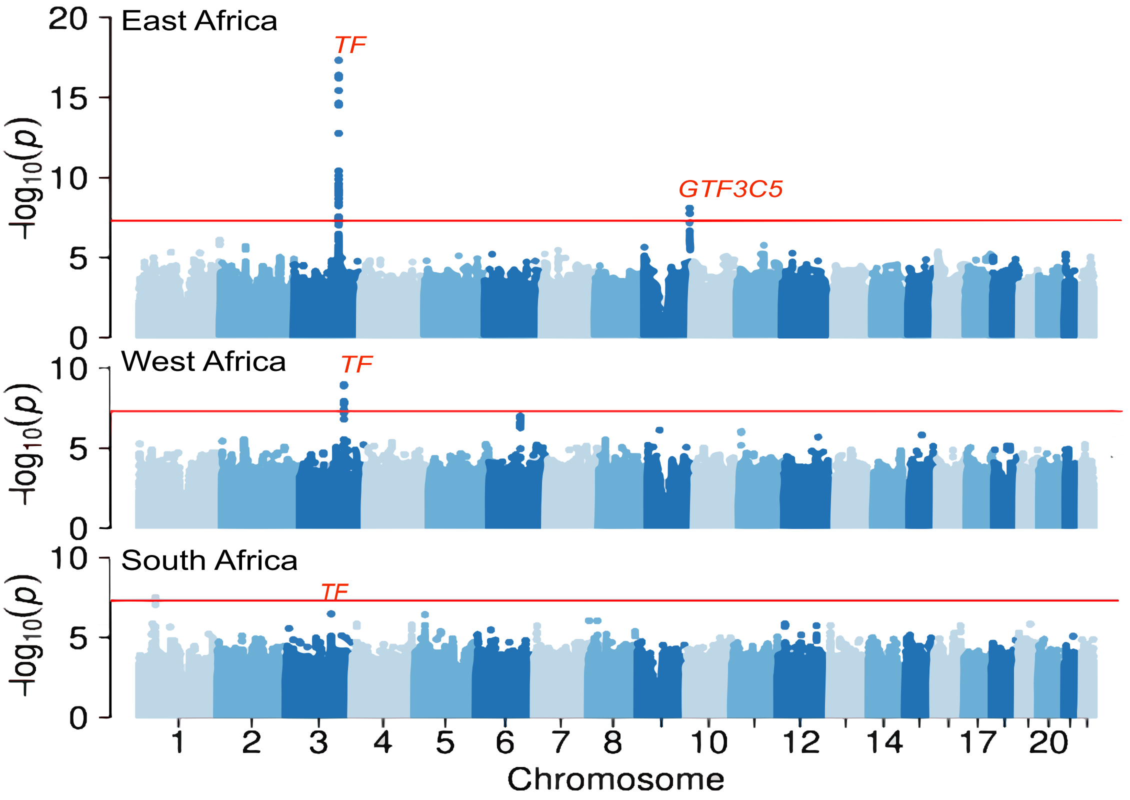

Supplementary Figure 3. Meta-analysis of transferrin GWAS results by African region. Genome-wide significant signals are annotated as *TF* and *GTF3C5* genes.

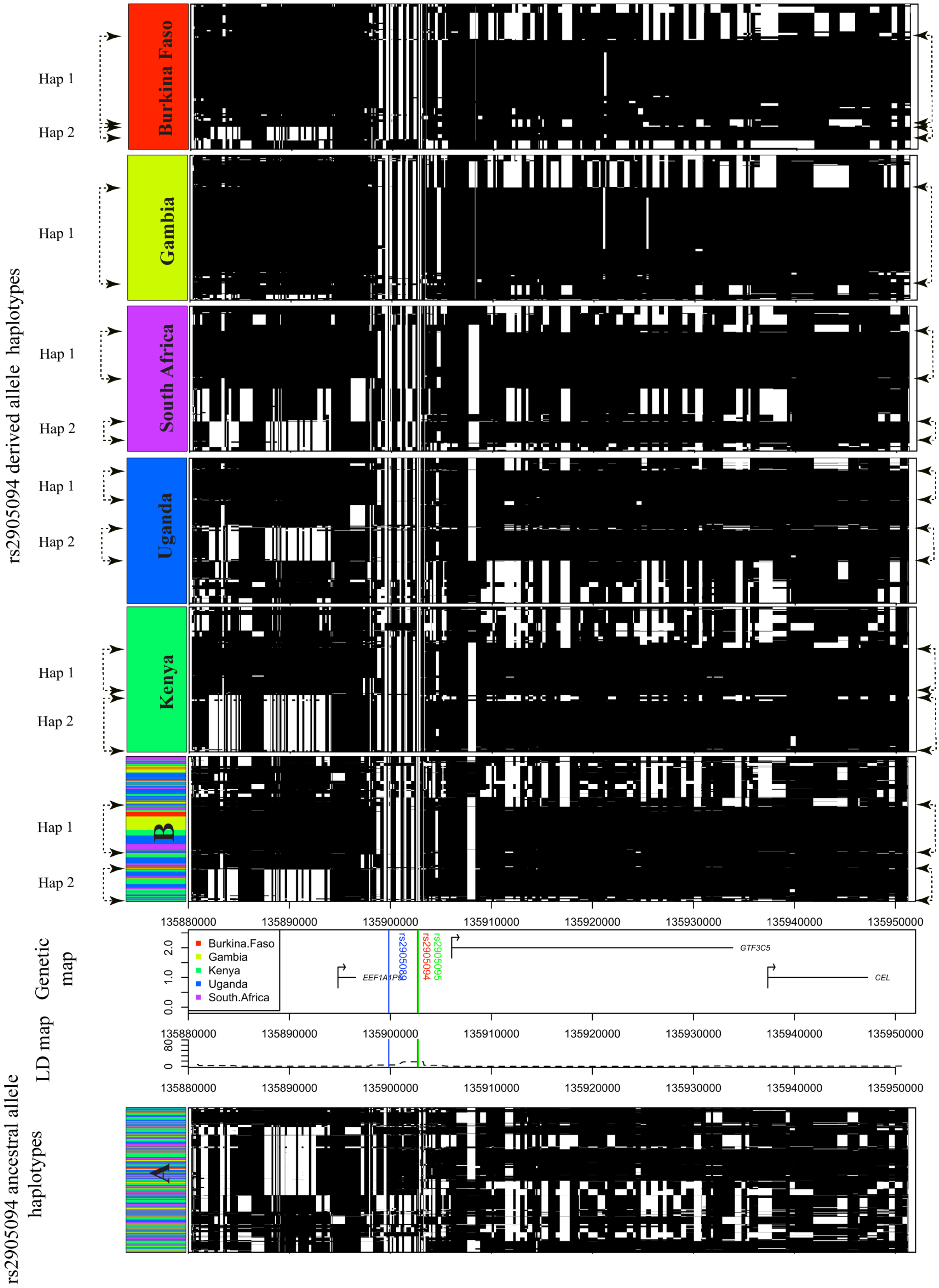

Supplementary Figure 4: Haplotype structures, linkage disequilibrium, and genetic map of the *GTF3C5* gene region. This figure presents the haplotype structures within a 72 KB region surrounding rs2905094 (9:135902689 (GRCh37)). **Panel A** displays the structure for the ancestral/reference allele, while **Panel B** illustrates the structure for the derived alleles across all countries combined. Additionally, haplotypes for the derived allele are shown

separately for each country, as labeled in the panels. In each panel, the rows represent individual phased haplotypes, and the columns denote SNPs, sorted and clustered based on Manhattan distance. The ancestral allele is depicted by black columns, and the derived allele is represented by white columns. The dominant haplotypes identified in the mutant chromosomes containing derived alleles are labelled as "Hap 1" and "Hap 2." The LD map is included with the Y-axis indicating the recombination rate in cM/Mb, and the X-axis displaying the chromosomal position referenced to GRCh37. The genetic map highlights the positions of three genome-wide significant SNPs - rs2905089, rs2905094, and rs2905095 - using blue, red, and green lines, respectively.

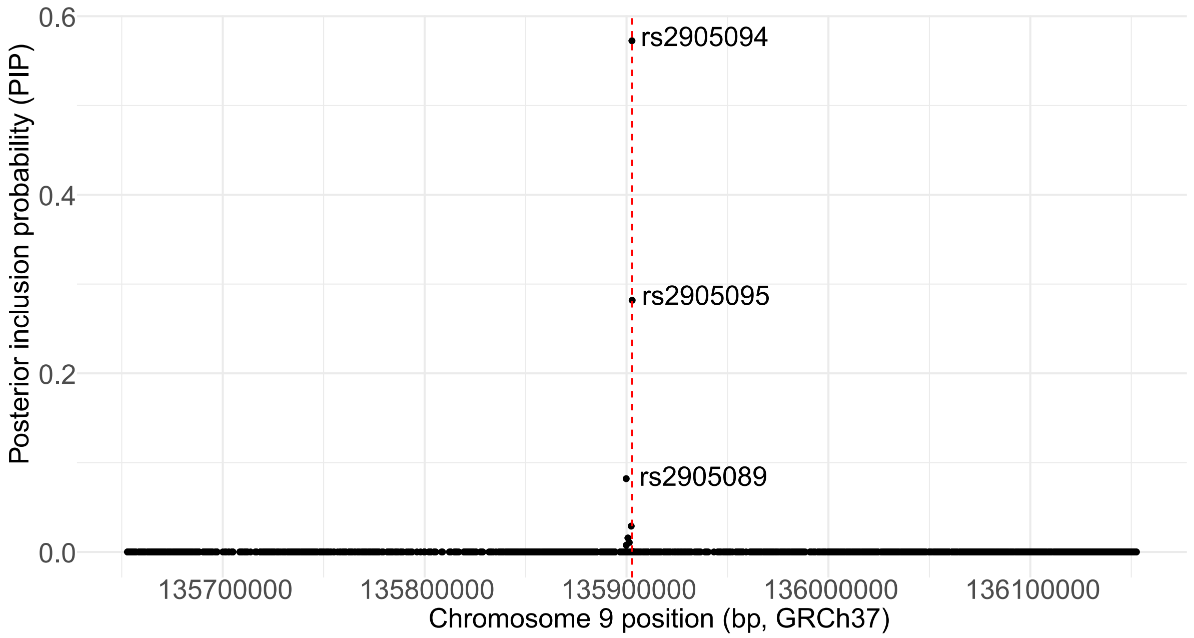

Supplementary Figure 5: Fine-mapping of *GTF3C5* locus in East African populations. Fine-mapping analysis was performed with *susieR* package using summary statistics from the meta-analysis of the Kenyan and Ugandan cohorts to estimate the posterior inclusion probability (PIP) for each variant within the *GTF3C5* locus.

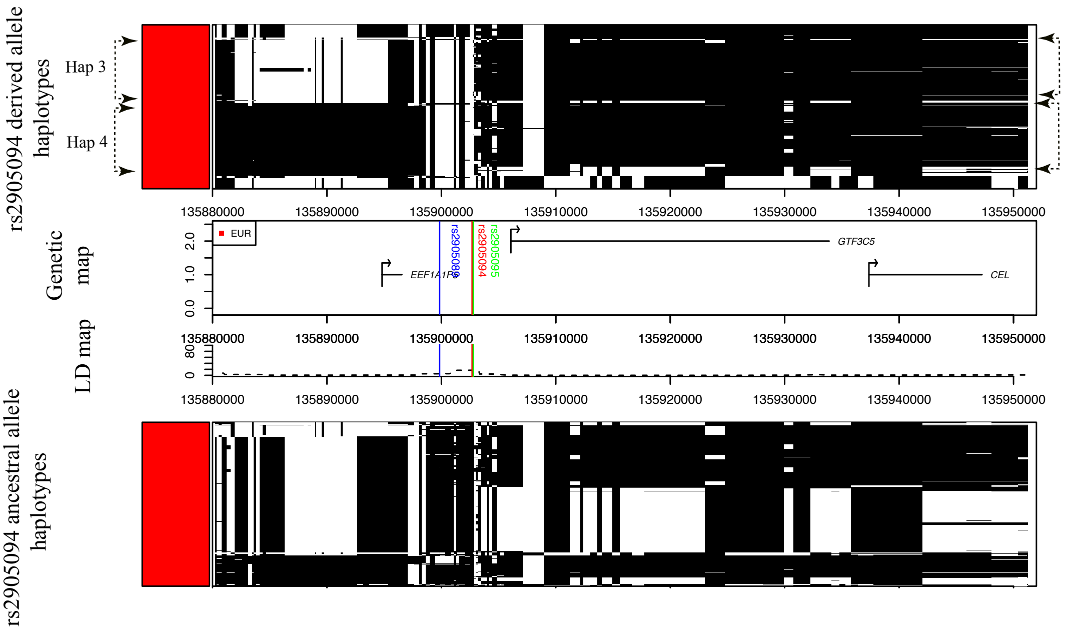

Supplementary Figure 6: Haplotype structure of the *GTF3C5* gene region in European 1000G data. This figure presents the haplotype structures within a 72 KB region surrounding rs2905094. The upper panel illustrates the structure for the derived allele (T) in European populations, while the lower panel displays the structure for the ancestral allele (C). Rows represent individual phased haplotypes, and the columns denote SNPs, sorted and clustered based on Manhattan distance. The ancestral allele is depicted by black columns, and the derived allele is represented by white columns. The dominant haplotypes identified in the mutant chromosomes containing derived alleles are labelled as "Hap 3" and "Hap 4." The LD map is included with the Y-axis indicating the recombination rate in cM/Mb, and the X-axis displaying the chromosomal position referenced to GRCh37. The genetic map highlights the positions of three genome-wide significant SNPs - rs2905089, rs2905094, and rs2905095 - using blue, red, and green lines, respectively.

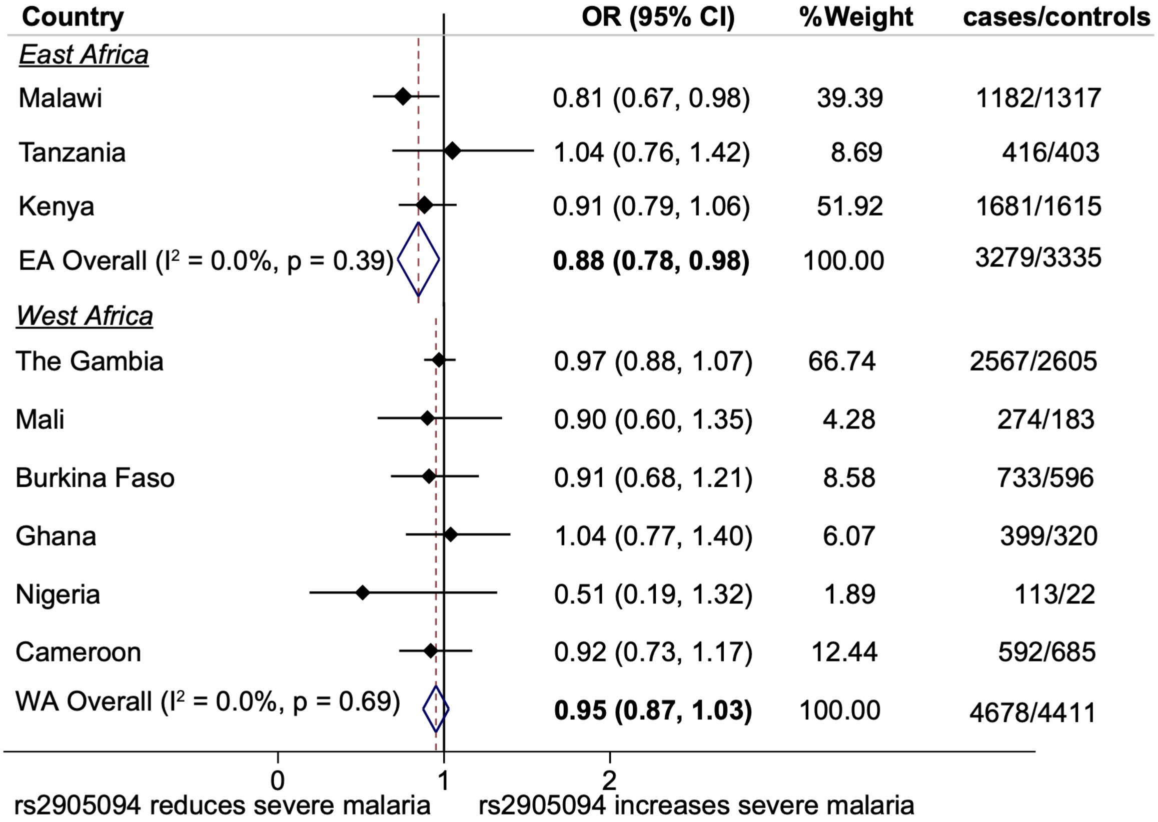

Supplementary Figure 7: Meta-analyses of the effect sizes of the *GTF3C5* lead SNP, rs2905094 and severe malaria. We applied fixed effects meta-analysis of severe malaria GWAS results from the MalariaGen consortium stratified by East (EA) and West African (WA) countries.[1] Odds ratio (center) and 95% confidence intervals (error bars) are shown.

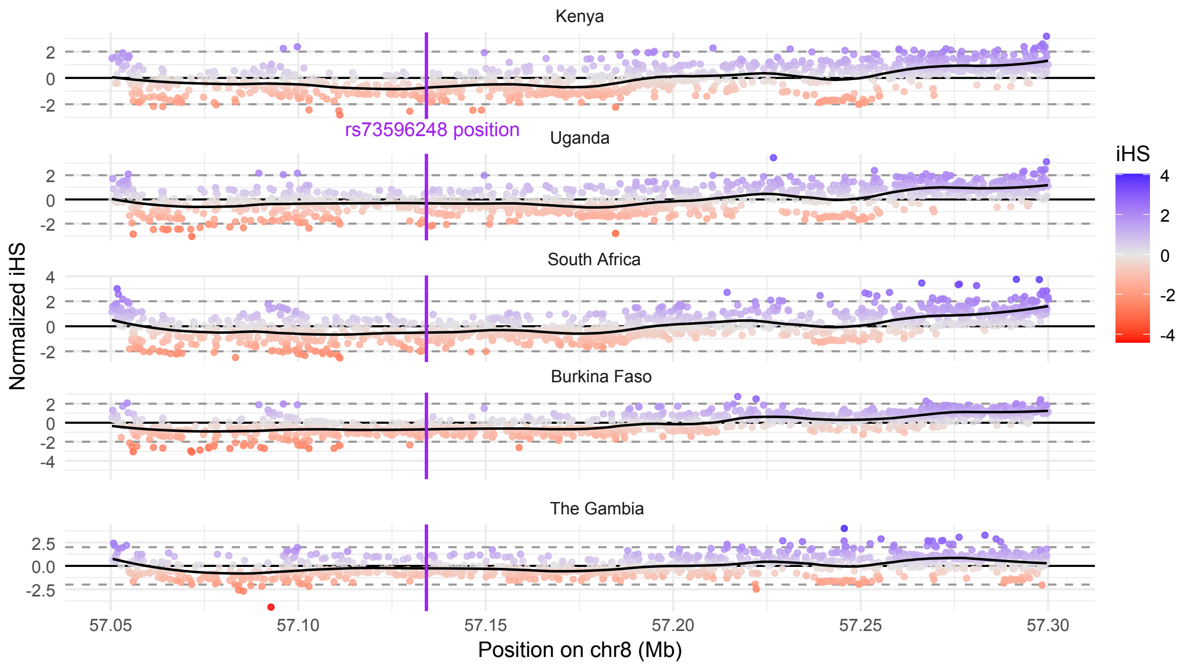

Supplementary Figure 8: Normalized integrated haplotype score (iHS) across chr8:57.05 - 57.30 Mb within *CHCHD7/SDR16C5* locus. iHS were computed using selscan v2.1.0.[2] SNPs with MAF ≥0.01 were included. Raw iHS were standardized by allele frequency bins (n=10) across the genome to account for variation in allele frequencies. Points are per-SNP normalized iHS; black line indicates loess fit.

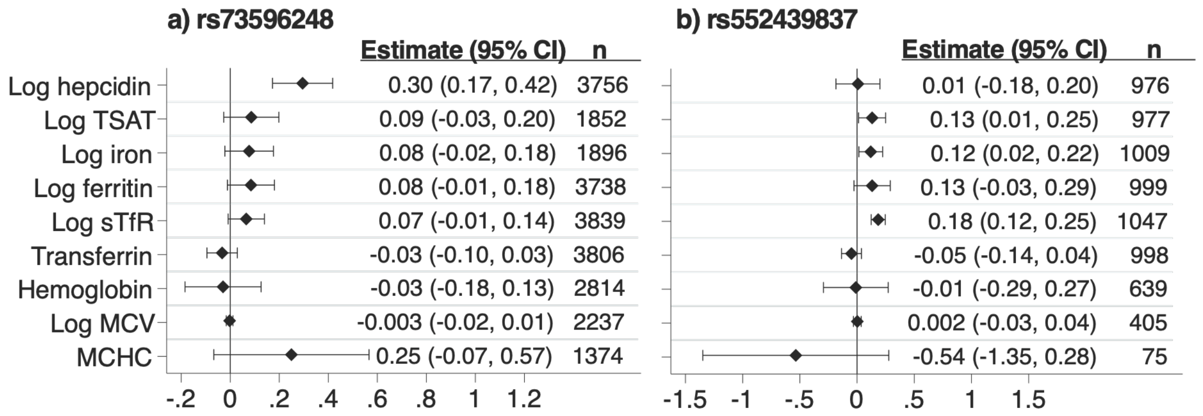

Supplementary Figure 9: Association between a) hepcidin and b) soluble transferrin receptor GWAS lead SNPs and iron biomarkers. Estimates (center), 95% confidence intervals (error bars) were derived from an additive linear regression model adjusted for age, sex, and study site. Since rs73596248 appeared across all five iron GWAS African sites, analyzed data included all five study sites while for rs552439837 data were from Kenya only. n indicates biologically independent samples. MCV, Mean Corpuscular Volume; MCHC, Mean Corpuscular Hemoglobin Concentration.

Supplementary Tables

Supplementary Table 1. Characteristics of study participants

Characteristic	Kenya n=1059		Uganda n=1360		Burkina Faso n=348		South Africa n=611		The Gambia n=550		Jackson Heart Study n=2868	
Median age (IQR)[a]	21.8 m (15.2-40.0)		24.1 m (23.9-35.9)		23.3 m (19.7-26.2)		12.0 m (11.9-12.2)		46.0 m (34.4-58.5)		54 y (44-64)	
	**n/total**	**%**	**n/total**	**%**	**n/total**	**%**	**n/total**	**%**	**n/total**	**%**	**n/total**	**%**
Gender: Females	514/1059	48.5	675/1360	49.6	173/348	49.7	294/611	48.1	250/550	45.5	1759/2868	61.3
Inflammation[b]	275/1020	27.0	314/1325	23.7	112/330	33.9	95/611	15.6	78/550	14.2	830/2864	29.0
Malaria parasitemia[c]	215/954	22.5	19/1340	6.8	66/321	20.6	0/611	0.0	59/548	10.8	0/2868	0.0
Iron deficiency[d]	350/999	35.0	436/1254	34.8	115/324	35.5	251/611	41.1	118/550	21.5	298/2868	10.4
Anemia[e]	420/639	65.7	646/1299	49.7	288/331	87.0	n/a	n/a	329/545	60.4	728/2818	25.8
Iron deficiency anemia[f]	140/609	23.0	253/1197	21.1	96/309	31.1	n/a	n/a	85/545	15.6	174/2142	8.1
Stunting[g]	89/183	48.6	207/1342	15.4	109/325	33.5	n/a	n/a	167/438	38.1	n/a	n/a
Underweight[h]	95/334	28.4	112/1355	8.3	60/327	18.4	n/a	n/a	111/439	25.3	n/a	n/a
Wasting[i]	24/180	13.3	66/1340	4.9	22/325	6.8	n/a	n/a	36/438	8.2	n/a	n/a
**Biomarker**	**n**	**GMean (SD)**	**n**	**GMean (SD)**	**n**	**GMean (SD)**	**n**	**GMean (SD)**	**n**	**GMean (SD)**	**n**	**GMean (SD)**
Ferritin, µg/L	999	21.9 (3.1)	1254	20.9 (2.9)	324	22.2 (2.9)	611	14.9 (2.6)	550	24.4 (2.4)	2868	112.2 (2.6)
sTfR, mg/L	1047	17.8 (1.5)	1330	6.7 (2.0)	342	17.7 (1.7)	610	10.8 (1.5)	510	3.5 (1.4)	n/a	n/a
Hepcidin, µg/L	976	5.7 (3.6)	1321	6.8 (3.3)	309	5.3 (4.2)	600	7.8 (3.5)	550	5.5 (4.7)	n/a	n/a
Serum iron, µmol/L	1009	6.5 (2.0)	n/a	n/a	337	6.0 (1.8)	n/a	n/a	550	8.6 (1.6)	2868	74.1 (1.5)
Transferrin, g/L	998	2.7 (1.3)	1320	2.7 (1.3)	327	2.7 (1.3)	611	2.6 (1.3)	550	2.6 (1.2)	2868	2.0 (1.2)
TSAT, %[j]	977	9.4 (2.2	n/a	n/a	325	8.9 (2.0)	n/a	n/a	550	12.8 (1.7)	2868	25.7 (1.5)
Hemoglobin, g/dL	639	10.2 (1.2)	1299	10.8 (1.1)	331	9.5 (1.1)	n/a	n/a	545	10.6 (1.1)	2818	12.9 (1.1)
MCV	405	67.9 (9.1)	1292	71.5 (7.6)	n/a	n/a	n/a	n/a	540	75.6 (6.5)	n/a	n/a
MCHC	75	31.8 (1.3)	1299	32.9 (1.8)	n/a	n/a	n/a	n/a	n/a	n/a	n/a	n/a
Body mass index	243	15.1 (1.2)	1342	15.7 (1.1)	324	15.4 (1.2)	n/a	n/a	549	14.6 (1.1)	2864	31.2 (1.2)
Inflammatory marker[k]	1020	1.8 (5.2)	1325	1.4 (5.1)	330	2.8 (6.1)	611	0.9 (5.0)	550	0.4 (1.3)	2864	2.5 (3.5)

IQR, interquartile range; n/a, not available; sTfR, soluble transferrin receptors; TSAT, transferrin saturation; CRP, C-reactive protein; MCV, Mean Corpuscular Volume; MCHC, Mean Corpuscular Hemoglobin Concentration; GMean, geometric means.

[a]Age in months for children and age in years for African American (Jackson Heart Study) adults.

[b]Inflammation was defined as C-reactive protein > 5mg/L or α1-antichymotrypsin > 0.6g/dL (in The Gambia). Inflammatory marker values indicate CRP (mg/L) for Kenya, Uganda, Burkina Faso and South Africa, and ACT (g/dL) for The Gambia.

[c]Malaria parasitemia was defined as *Plasmodium falciparum* parasitemia positive at any density. Participants from South Africa and the Jackson Heart Study were not tested for malaria since malaria is not endemic in these regions.

[d]Iron deficiency defined using WHO definition as ferritin < 12 µg/L or <30 µg/L in presence of inflammation (C-reactive protein>5mg/L or α1-antichymotrypsin >0.6g/dL) in children < 5 years or <15µg/L in children >=5 years. In the Jackson Heart Study iron deficiency was defined as ferritin levels <30 µg/L

[e]Anemia was defined as hemoglobin <11 g/dL in children aged <5 years, or hemoglobin <11.5 g/dL in children ≥5 years[3]. In the Jackson Heart Study, ID was defined as ferritin <30µg/L. In the Jackson Heart Study, anemia was defined as hemoglobin <12 g/dL in women or <13 g/dL in men[3,4].

[f]Iron deficiency anemia was defined as iron deficiency and anemia.

[g]Stunting was defined as height-for-age z-score < -2

[h]Underweight was defined as weight-for-age z-score < -2

[i]Wasting was defined as weight-for-height z-score < -2

[j]TSAT was calculated as ((Iron (µmol/L)/Transferrin (g/L) x 25.1) x 100) except in The Gambia where it was calculated using iron and unsaturated iron binding capacity. Iron and TSAT were missing in Uganda and South Africa, and transferrin was missing in The Gambia.

[k]Inflammatory markers included CRP (mg/L) measured in Kenya, Uganda, Burkina Faso, South Africa, and Jackson Heart Study and ACT (g/dL) measured in The Gambia.

Supplementary Table 2. Summary of quality control (QC) steps for each population

	Kenya	Uganda	Burkina Faso	South Africa	The Gambia	The Gambia	JHS
Pre-QC							
Total number sent for genotyping	1,312	1,429	354	777	528	46	3,021
Number of variants typed and mapping to Build 37	2,114,814	2,337,579	2,337,579	2,337,579	2,108,163	2,114,814	874,712
Individuals QC							
Individuals with missingness >0.03	3	14	0	2	0	0	135
Individuals with extreme heterozygosity (>0.343 or <0.15)	0	0	0	0	0	0	0
Individuals failing sex- check	58	3	0	2	13	0	4
Individuals IBD>0.9 and not twins / triplets	32	22	0	24	9	2	3
Autosomal variants QC							
Non-autosomal variants	36,413	51,101	51,101	51,101	36,328	36,413	34,771
Autosomal duplicate variants	28	9,248	9,248	9,248	26	28	2,830
Autosomal variants with missingness>0.01	93,959	262,676	263,935	226,503	70,238	89,922	122,182
Autosomal variants with MAF<0.01	211,678	392,392	399,486	416,608	224,200	231,339	342
Autosomal variants in Hardy-Weinberg disequilibrium (P<0.008)	29,735	29,266	20,089	25,174	22,471	6,544	17,258
Post-QC							
Total individuals remaining	1,219	1,390	354	749	506	44	2,879
Total autosomal variants remaining	1,743,001	1,592,896	1,593,720	1,608,945	1,754,900	1,750,568	697,329

The number of individuals and variants removed within each population are presented. Genotyping for the 574 and 46 Gambian samples was done separately and then merged after quality control giving total individuals remaining=550 and total autosomal variants remaining 1,905,494. JHS, Jackson Heart Study.

Supplementary Table 3. Number of variants imputed into individual datasets

Cohort	Total imputed	Total high quality	Total tested for association
Kenya	89,838,087	13,870,649	7,756,141
Uganda	89,838,087	13,982,934	13,765,898
Burkina Faso	89,838,087	13,128,631	13,042,768
South Africa	89,838,087	15,010,497	14,457,588
The Gambia	89,838,087	12,770,741	10,295,094
Jackson Heart Study	89,838,087	13,455,566	13,349,077

High quality SNPs are those only with an information score greater than 0.3 and those tested for association were only those of high quality with a minor allele frequency (MAF) greater than 0.01.

Supplementary Table 4. Comparison of results adjusted for age and sex and those additionally adjusted for inflammatory markers for top independent SNPs associated with iron biomarkers in African children

							Age and sex adjusted			Age, sex and inflammation adjusted		
**SNP**	**CHR**	**Hg19 BP**	**EA**	**OA**	**Gene**	**EA Freq**	**Beta**	**SE**	**P**	**Beta**	**SE**	**P**
Transferrin (all African sites)												
rs6762719	3	133480817	G	A	*TF*	0.25	0.28	0.03	1.59×10^{-27}	0.28	0.03	1.78×10^{-21}
rs4854748	3	133446881	T	C	*TF*	0.20	-0.24	0.03	1.12×10^{-16}	-0.25	0.03	6.24×10^{-15}
Transferrin (Uganda and Kenya)												
rs2905094	9	135902689	T	C	*GTF3C5*	0.16	-0.23	0.04	8.08×10^{-9}	-0.22	0.04	1.86×10^{-8}
Hepcidin (all African sites)												
rs73596248	8	57134187	A	G	*CHCHD7/SDR16C5*	0.06	0.27	0.05	4.51×10^{-8}	0.26	0.05	1.85×10^{-8}
sTfR (Kenya)												
rs552439837	4	144446525	G	A	*SMARCA5*	0.09	0.48	0.09	2.78×10^{-8}	0.47	0.09	3.51×10^{-8}

CHR, chromosome; Hg19 BP, human genome build 19 base pair; EA, effect allele; OA, other allele; EA Freq, effect allele frequency; SE, standard error; P, two-sided GWAS P value from an additive model.

Supplementary Table 5. Genome-wide significant SNPs associated with transferrin levels at *GTF3C5* locus in meta-analysis of Kenya and Uganda and how they compare with other populations

	**SNP**	**rs2905094**	**rs2905095**	**rs2905089**
	CHR	9	9	9
	Hg19BP	135902689	135902793	135899844
	EA	T	T	C
	OA	C	C	A
	Consequence	upstream gene variant	upstream gene variant	upstream gene variant
Kenya+Uganda meta-analysis	EA Freq	0.16	0.18	0.25
	Beta	-0.23	-0.22	-0.18
	SE	0.04	0.04	0.03
	P	8.08E-09	1.75E-08	6.74E-08
Uganda	EA Freq	0.18	0.20	0.29
	Beta	-0.20	-0.19	-0.18
	SE	0.05	0.05	0.04
	P	9.82E-05	9.39E-05	3.60E-05
Kenya	EA Freq	0.14	0.16	0.21
	Beta	-0.29	-0.26	-0.20
	SE	0.07	0.06	0.06
	P	1.15E-05	3.17E-05	4.94E-04
South Africa	EA Freq	0.14	0.16	0.25
	Beta	-0.15	-0.13	-0.10
	SE	0.08	0.08	0.07
	P	7.35E-02	9.95E-02	1.59E-01
The Gambia	EA Freq	0.23	0.25	0.32
	Beta	0.12	0.06	-0.04
	SE	0.07	0.07	0.06
	P	1.06E-01	3.55E-01	5.81E-01
Burkina Faso	EA Freq	0.17	0.18	0.25
	Beta	-0.03	-0.06	0.09
	SE	0.10	0.09	0.09
	P	7.86E-01	5.08E-01	3.02E-01
Jackson Heart Study	EA Freq	0.18	0.19	0.27
	Beta	-0.02	-0.03	-0.02
	SE	0.04	0.04	0.03
	P	5.78E-01	4.62E-01	5.43E-01
European Ancestry Meta-analysis[a]	EA Freq	0.33	0.30	0.33
	Beta	0.004	0.01	0.001
	**SE**	0.004	0.007	0.004
	**P**	3.20E-01	1.10E-01	7.40E-01

CHR, chromosome; Hg19BP, human build 19 base pair; EA, effect allele; OA, other allele; EA Freq, effect allele frequency; SE, standard error; P, additive model GWAS p value
[a]Effect of SNPs on transferrin iron binding capacity reported by Moksnes et al 2022[5] in European ancestry participants.

Supplementary Table 6. Distribution of haplotypes carrying the rs2905094-T allele at the *GTF3C5* locus across African populations

Population	Haplotype 1, n (%)	Haplotype 2, n (%)	Other n (%)
Kenya	109 (32.2)	113 (33.3)	117 (34.5)
Uganda	115 (23.0)	100 (20.0)	285 (57.0)
South Africa	66 (31.3)	31 (14.7)	114 (54.0)
Burkina Faso	61 (52.1)	10 (8.6)	46 (39.3)
The Gambia	151 (62.1)	0 (0)	92 (37.9)

Supplementary Table 7. *GTF3C5* haplotype-level association analysis in East Africa

	Joint			Hap1-only			Hap2-only			rs2905094 conditional			Among T carriers		
	Beta	SE	P	Beta	SE	P	Beta	SE	P	Beta	SE	P	Beta	SE	P
Haplotype 1	-0.256	0.070	0.0003	-0.251	0.070	0.0004				-0.029	0.086	0.732	-0.048	0.086	0.576
Haplotype 2	-0.154	0.071	0.030				-0.146	0.071	0.039	0.068	0.085	0.426	0.054	0.087	0.535
Age in years	-0.164	0.016	2.67E-25	-0.164	0.016	2.97E-25	-0.163	0.016	5.80E-25	-0.162	0.016	6.75E-25	-0.118	0.030	0.0001
Sex: Female	-0.069	0.041	0.089	-0.068	0.041	0.094	-0.073	0.041	0.074	-0.078	0.041	0.054	-0.050	0.075	0.502
Country: Uganda	-0.009	0.041	0.830	-0.006	0.041	0.885	-0.007	0.041	0.875	0.017	0.041	0.682	0.120	0.077	0.122
PC1	3.585	0.737	1.23E-06	3.625	0.739	9.85E-07	3.478	0.736	2.48E-06	3.964	0.735	7.67E-08	3.844	1.230	0.002
PC2	-2.135	0.802	0.008	-2.118	0.803	0.008	-2.270	0.799	0.005	-2.116	0.796	0.008	-1.041	1.642	0.526
PC3	1.374	0.614	0.025	1.407	0.619	0.023	1.328	0.612	0.030	1.548	0.616	0.012	0.316	2.791	0.910
PC4	1.828	0.654	0.005	1.840	0.660	0.005	1.811	0.660	0.006	1.833	0.628	0.004	-0.195	2.302	0.932
PC5	1.009	0.612	0.099	0.966	0.614	0.116	1.058	0.620	0.088	0.951	0.609	0.119	1.038	2.470	0.675
PC6	0.593	0.700	0.397	0.548	0.697	0.432	0.797	0.683	0.244	0.530	0.692	0.444	0.209	1.135	0.854
PC7	0.594	0.854	0.487	0.571	0.858	0.506	0.590	0.854	0.490	0.624	0.855	0.466	0.249	1.791	0.890
PC8	0.016	0.586	0.979	-0.012	0.594	0.984	0.115	0.594	0.846	0.165	0.570	0.773	1.294	1.483	0.383
PC9	-0.981	0.749	0.190	-1.071	0.754	0.155	-1.015	0.749	0.176	-0.929	0.734	0.205	-0.990	1.756	0.573
PC10	-0.529	0.767	0.491	-0.551	0.764	0.471	-0.492	0.767	0.521	-0.581	0.761	0.446	-1.453	1.964	0.460
rs2905094										-0.248	0.054	5.49E-06			

Haplotype dosages were encoded per individual as counts (0/1/2) for Haplotype 1 and Haplotype 2. Linear regression of normalized transferrin levels was performed with robust standard errors, adjusting for age, sex, country and principal components 1 to 10. We ran joint models with haplotype 1 and haplotype 2 together, haplotype-only models, and models conditioned on rs290504 genotype (all n=2318 independent samples); we additionally restricted analyses to carriers of the rs2905094-T allele (n=697). SE, standard error; P, two-tailed p-value.

Supplementary Table 8. Summary statistics for bacteremia and severe malaria GWAS at lead African-specific iron-related SNPs

	SNP	rs2905094			rs73596248			rs552439837*		
	Genotype	CC	CT	TT	GG	GA	AA	AA	AG	GG
1	**Bacteremia**	1477 (33.5%)	466 (31.6%)	27 (28.7%)	1757 (33.4%)	199 (29.0%)	14 (42.4%)	1308 (37.2%)	224 (33.6%)	4 (12.5%)
	Escherichia coli	157 (3.6%)	49 (3.3%)	3 (3.2%)	191 (3.6%)	18 (2.6%)	0 (0.0%)	139 (4.0%)	24 (3.6%)	0 (0.0%)
	Klebsiella pneumoniae	49 (1.1%)	20 (1.4%)	0 (0.0%)	67 (1.3%)	2 (0.3%)	0 (0.0%)	36 (1.0%)	9 (1.4%)	0 (0.0%)
	Non-typhoidal *Salmonella*	163 (3.7%)	46 (3.1%)	2 (2.1%)	188 (3.6%)	21 (3.1%)	2 (6.1%)	150 (4.3%)	23 (3.5%)	1 (3.1%)
	Staphylococcus aureus	165 (3.7%)	55 (3.7%)	4 (4.3%)	207 (3.9%)	17 (2.5%)	0 (0.0%)	153 (4.4%)	25 (3.8%)	0 (0.0%)
	Hemophilus influenzae	126 (2.9%)	36 (2.4%)	2 (2.1%)	149 (2.8%)	14 (2.0%)	1 (3.0%)	115 (3.3%)	17 (2.6%)	1 (3.1%)
	Streptococcus pneumoniae	387 (8.8%)	143 (9.7%)	7 (7.5%)	464 (8.8%)	67 (9.8%)	6 (18.2%)	353 (10.0%)	69 (10.4%)	1 (3.1%)

2 Severe malaria

SNP	Iron phenotype	Country	Cases	Controls	n	MAF	Beta	SE	P
rs2905094	Transferrin	Malawi	1182	1317	2499	0.099	-0.213	0.098	0.029
rs2905094	Transferrin	Tanzania	416	403	819	0.122	0.045	0.157	0.775
rs2905094	Transferrin	Kenya	1681	1615	3296	0.144	-0.088	0.074	0.237
rs73596248	Hepcidin	The Gambia	2567	2605	5172	0.059	0.039	0.088	0.652
rs73596248	Hepcidin	Mali	274	183	457	0.064	-0.184	0.286	0.520
rs73596248	Hepcidin	Burkina Faso	733	596	1329	0.066	0.203	0.219	0.351
rs73596248	Hepcidin	Ghana	399	320	719	0.086	-0.160	0.194	0.407
rs73596248	Hepcidin	Nigeria	113	22	135	0.086	0.651	0.772	0.362
rs73596248	Hepcidin	Cameroon	592	685	1277	0.082	0.014	0.153	0.928
rs73596248	Hepcidin	Malawi	1182	1317	2499	0.074	-0.105	0.110	0.339
rs73596248	Hepcidin	Tanzania	416	403	819	0.065	-0.128	0.204	0.530
rs73596248	Hepcidin	Kenya	1681	1615	3296	0.065	0.047	0.106	0.660
rs141274959	sTfR	Kenya	1681	1615	3296	0.075	-0.578	0.104	3.3×10^{-9}

In the bacteremia GWAS,[6] numbers are cases and their percentage per SNP genotype. *rs552439837 was missing in the replication GWAS data. Severe malaria GWAS data are from the MalariaGen Consortium.[1] n=biologically independent samples. MAF=minor allele frequency. Beta=additive effect (log odds). SE=standard error. P = additive model two-tailed p-value.

Supplementary Table 9. Top SNPs associated with hepcidin levels in continental African populations and their frequencies in European, Asian and Latin American populations

					Continental Africa						European ancestry	Asian ancestry	Latin American ancestry
CHR	SNP	Hg19BP	EA	OA	Consequence	Nearest gene	EA Freq	Beta	SE	P	EAfreq	EAfreq	EAfreq
8	rs73596248	57134187	A	G	intergenic	*CHCHD7*	0.06	0.27	0.05	4.51E-08	0.00035	0.00	0.006
8	rs59493383	57199697	G	T	intergenic	Not listed	0.06	0.27	0.05	6.63E-08	0.0003	0.00	0.014
8	rs73596251	57135454	C	T	downstream_gene_variant	*CHCHD7*	0.06	0.27	0.05	6.64E-08	0.0003	0.00	0.007
8	rs57580475	57199467	G	C	intergenic	Not listed	0.06	0.27	0.05	8.78E-08	0.0003	0.00	0.014
8	rs73596246	57133643	C	G	downstream_gene_variant	*CHCHD7*	0.06	0.27	0.05	4.51E-08	0.0003	0.00	0.007

CHR, chromosome; Hg19BP, human build 19 base pair; EA, effect allele; OA, other allele; EAFreq, effect allele frequency; SE, standard error; P, additive model GWAS p-value. Africans includes meta-analyzed results from the five continental African sites included in this study. Frequencies for European, Asian and Latin American populations are from dbSNP database (https://www.ncbi.nlm.nih.gov/snp) Allele Frequency Aggregator (ALFA).

Supplementary Table 10. Top SNPs associated with soluble transferrin receptor levels in Kenya and how they compare with other continental African populations

							Kenya				Uganda				South Africa				Burkina Faso			
SNP[a]	CHR	Hg19BP	EA	OA	Consequence	Nearest Gene	EAFreq	Beta	SE	P	EAFreq	Beta	SE	P	EAFreq	Beta	SE	P	EAFreq	Beta	SE	P
rs552439837	4	144446525	G	A	intron_variant	*SMARCA5*	0.09	0.48	0.09	2.78E-08	Missing	NA	NA	NA	Missing	NA	NA	NA	Missing	NA	NA	NA
rs573724761	4	144449330	G	C	intron_variant	*SMARCA5*	0.09	0.48	0.09	2.78E-08	Missing	NA	NA	NA	Missing	NA	NA	NA	Missing	NA	NA	NA
rs141274959	4	144587704	A	G	intron_variant	*FREM3*	0.07	0.56	0.10	6.00E-08	Missing	NA	NA	NA	Missing	NA	NA	NA	Missing	NA	NA	NA
rs1040736918	4	144961711	T	C	intron_variant	*GYPB*	0.06	0.74	0.14	6.15E-08	Missing	NA	NA	NA	Missing	NA	NA	NA	Missing	NA	NA	NA
rs554867097	4	145087102	G	A	Intergenic	Not listed	0.06	0.57	0.11	6.22E-08	Missing	NA	NA	NA	0.02	-0.01	0.22	0.97	Missing	NA	NA	NA
rs113319340	4	144462931	G	T	intron_variant	*SMARCA5*	0.10	0.43	0.08	7.66E-08	Missing	NA	NA	NA	Missing	NA	NA	NA	Missing	NA	NA	NA
rs563199246	4	144504069	A	G	intron_variant	*FREM3*	0.09	0.46	0.09	7.96E-08	Missing	NA	NA	NA	Missing	NA	NA	NA	Missing	NA	NA	NA
rs111374053	4	144501489	T	C	intron_variant	*FREM3*	0.09	0.45	0.08	8.17E-08	Missing	NA	NA	NA	Missing	NA	NA	NA	Missing	NA	NA	NA
rs34330779	4	145124084	A	G	intron_variant	Not listed	0.09	0.42	0.08	9.01E-08	Missing	NA	NA	NA	0.02	-0.34	0.20	0.09	0.01	0.02	0.33	0.94
rs535317699	4	144532466	C	G	Synonymous	*FREM3*	0.08	0.52	0.10	9.28E-08	Missing	NA	NA	NA	Missing	NA	NA	NA	NA	NA	NA	NA

CHR, chromosome; Hg19BP, human build 19 base pair; EA, effect allele; OA, other allele; EAFreq, effect allele frequency; SE, standard error; P, additive model GWAS p-value; NA, not applicable

[a]These SNPs were monomorphic in The Gambia, JHS, and in European populations.

Supplementary Table 11. Laboratory assays for iron and inflammatory biomarkers by site

Study site	Transferrin	Ferritin	sTfR	Hepcidin	Iron	UIBC	TIBC	CRP	ACT
Kilfi, Kenya	Chemiluminescent Microparticle Immunoassay (Abbott Architect, USA)	Microparticle Enzyme Immunoassay (Abbott Architect, USA)	Human sTfR ELISA (BioVendor, Czech Republic)	DRG Hepcidin 25 [bioactive] high sensitive ELISA (DRG International, USA)	MULTIGENT iron calorimetric assay, Abbott Architect, USA	Not analyzed	Not analyzed	MULTIGENT CRP Vario assay, (Abbott Architect, USA)	Not analyzed
Banfora,Burkina Faso	Chemiluminescent Microparticle Immunoassay (Abbott Architect, USA)	Microparticle Enzyme Immunoassay (Abbott Architect, USA)	Human sTfR ELISA (BioVendor, Czech Republic)	DRG Hepcidin 25 [bioactive] high sensitive ELISA (DRG International, USA)	MULTIGENT iron calorimetric assay, Abbott Architect, USA	Not analyzed	Not analyzed	MULTIGENT CRP Vario assay, (Abbott Architect, USA)	Not analyzed
Entebbe, Uganda	Chemiluminescent Microparticle Immunoassay (Abbott Architect, USA)	Microparticle Enzyme Immunoassay (Abbott Architect, USA)	Human sTfR ELISA (BioVendor, Czech Republic)	DRG Hepcidin 25 [bioactive] high sensitive ELISA (DRG International, USA)	Not analyzed	Not analyzed	Not analyzed	MULTIGENT CRP Vario assay, (Abbott Architect, USA)	Not analyzed
Soweto, South Africa	Chemiluminescent Microparticle Immunoassay (Abbott Architect, USA)	Microparticle Enzyme Immunoassay (Abbott Architect, USA)	Human sTfR ELISA (BioVendor, Czech Republic)	DRG Hepcidin 25 [bioactive] high sensitive ELISA (DRG International, USA)	Not analyzed	Not analyzed	Not analyzed	MULTIGENT CRP Vario assay, (Abbott Architect, USA)	Not analyzed
West Kiang, The Gambia	Not analyzed	Microparticle Enzyme Immunoassay (Abbott Architect, USA)	Quantikine sTfR ELISA kit, (R&D Systems, USA)	Hepcidin-25 [human] Enzyme Immunoassay Kit (Bachem, Switzerland)	Ferrozine-based photometry and colorimetry analyser (Hitachi 911, Hitachi, Tokyo, Japan)	Ferrozine-based photometry and colorimetry analyser (Hitachi 911, Hitachi, Tokyo, Japan)	Not analyzed	Not analyzed	Immunoturbidimetry, Cobas Mira Plus Bio-analyser, Roche
Jackson Heart Study	Not analyzed	Roche immunoturbimetric assay	Not analyzed	Not analyzed	Ferrozine colorimetric assay (Roche)	Not analyzed	Ferrozine colorimetric assay (Roche)	Immunoturbidim etric CRP-Latex assay (Roche)	Not analyzed

sTfR, soluble transferrin receptor; UIBC, unsaturated iron binding capacity; TIBC, total iron binding capacity; CRP, C-reactive protein; ACT, α1-antichymotrypsin

The Gambian hepcidin values were harmonized with the rest of the cohorts' hepcidin values by converting to the old DRG hepcidin assay values and then to the new high sensitive DRG hepcidin assay values.[7] We could not convert between the sTfR kits. Serum iron measurements were not done in Uganda and South Africa because plasma samples were stored in ethylenediaminetetraacetic acid (EDTA), which chelates iron.

Supplementary References

1. Malaria Genomic Epidemiology Network. Insights into malaria susceptibility using genome-wide data on 17,000 individuals from Africa, Asia and Oceania. *Nat. Commun.* **10**, 5732 (2019).
2. Szpiech, Z. A. Selscan 2.0: Scanning for sweeps in unphased data. *Bioinformatics* **40**, btae006 (2024).
3. World Health Organization. *Iron deficiency anaemia: assessment, prevention, and control. A guide for programme managers.* https://cdn.who.int/media/docs/default-source/2021-dha-docs/ida_assessment_prevention_control.pdf (2001).
4. Camaschella, C. Iron-deficiency anemia. *N. Engl. J. Med.* **372**, 1832–1843 (2015).
5. Moksnes, M. R. *et al.* Genome-wide meta-analysis of iron status biomarkers and the effect of iron on all-cause mortality in HUNT. *Commun. Biol.* **5**, 591 (2022).
6. The Kenyan Bacteraemia Study Group *et al.* Polymorphism in a lincRNA Associates with a doubled risk of pneumococcal bacteremia in Kenyan children. *Am. J. Hum. Genet.* **98**, 1092–1100 (2016).
7. Wray, K. *et al.* Hepcidin detects iron deficiency in Sri Lankan adolescents with a high burden of hemoglobinopathy: A diagnostic test accuracy study. *Am. J. Hematol.* **92**, 196–203 (2017).
