# Supplementary material for: African-specific genetic loci determine iron status and risk of severe malaria and bacteremia in African children
Source: Nat Commun. 2026 Apr 7;17:7068. doi: 10.1038/s41467-026-71567-w (PMC13392482; doi:10.1038/s41467-026-71567-w)
Supplement: Supplementary file 2 — Reporting summary [file 41467_2026_71567_MOESM2_ESM.pdf]

nature portfolio

Corresponding author(s): John Muthii Muriuki and Sarah Atkinson

Last updated by author(s): 12 March 2026

Reporting Summary

Nature Portfolio wishes to improve the reproducibility of the work that we publish. This form provides structure for consistency and transparency in reporting. For further information on Nature Portfolio policies, see our Editorial Policies and the Editorial Policy Checklist.

Statistics

For all statistical analyses, confirm that the following items are present in the figure legend, table legend, main text, or Methods section.

n/a	Confirmed	
☐	☒	The exact sample size (*n*) for each experimental group/condition, given as a discrete number and unit of measurement
☐	☒	A statement on whether measurements were taken from distinct samples or whether the same sample was measured repeatedly
☐	☒	The statistical test(s) used AND whether they are one- or two-sided<br>*Only common tests should be described solely by name; describe more complex techniques in the Methods section.*
☐	☒	A description of all covariates tested
☐	☒	A description of any assumptions or corrections, such as tests of normality and adjustment for multiple comparisons
☐	☒	A full description of the statistical parameters including central tendency (e.g. means) or other basic estimates (e.g. regression coefficient) AND variation (e.g. standard deviation) or associated estimates of uncertainty (e.g. confidence intervals)
☐	☒	For null hypothesis testing, the test statistic (e.g. *F*, *t*, *r*) with confidence intervals, effect sizes, degrees of freedom and *P* value noted<br>*Give P values as exact values whenever suitable.*
☒	☐	For Bayesian analysis, information on the choice of priors and Markov chain Monte Carlo settings
☒	☐	For hierarchical and complex designs, identification of the appropriate level for tests and full reporting of outcomes
☐	☒	Estimates of effect sizes (e.g. Cohen's *d*, Pearson's *r*), indicating how they were calculated

Our web collection on statistics for biologists contains articles on many of the points above.

Software and code

Policy information about availability of computer code

Data collection	Microsoft Excel 2016 for the phenotypes. Genotyping using HumanOmni 2.5M 8 Beadchip (in Uganda, South Africa and Burkina Faso) and H3Africa array (in Kenya and The Gambia).
Data analysis	R version 4.2.3. GCTA version 1.24.4. METASOFT version 2.0.1. Selscan V2.1.0.

For manuscripts utilizing custom algorithms or software that are central to the research but not yet described in published literature, software must be made available to editors and reviewers. We strongly encourage code deposition in a community repository (e.g. GitHub). See the Nature Portfolio guidelines for submitting code & software for further information.

Data

Policy information about availability of data

All manuscripts must include a data availability statement. This statement should provide the following information, where applicable:

- Accession codes, unique identifiers, or web links for publicly available datasets
- A description of any restrictions on data availability
- For clinical datasets or third party data, please ensure that the statement adheres to our policy

Summary statistics for the genome-wide association tests of imputed data for iron biomarkers in continental African populations reported in this study have been deposited in the Harvard Dataverse at https://doi.org/10.7910/DVN/ZGGN2F. The supplementary data generated in this study are provided in the Supplementary Information and Supplementary Data files. The European GWAS meta-analysis summary level data for serum ferritin, serum iron, total iron-binding capacity, and transferrin saturation used in this study are available from NTNU Open Research Data (https://doi.org/10.18710/S9TJEL), while those for hepcidin and soluble

transferrin receptors were obtained from the NHGRI-EBI GWAS Catalogue (hepcidin: accession number GCST90451683; soluble transferrin receptor: accession number GCST90451684).

Research involving human participants, their data, or biological material

Policy information about studies with human participants or human data. See also policy information about sex, gender (identity/presentation), and sexual orientation and race, ethnicity and racism.

Reporting on sex and gender	All analyses are based on self-reported sex which was confirmed using genetically determined sex.
Reporting on race, ethnicity, or other socially relevant groupings	The study reports primary data from children of continental African ancestry and African American adults, and secondary data from adults of European ancestry.
Population characteristics	The study includes children from five African countries and African American adults from the Jackson Heart Study and the details of population characteristics are provided in Supplementary Table 1. We also include results from published European iron GWAS studies and severe malaria and bacteremia GWAS and population characteristics from these studies appear in their original reports.
Recruitment	We used genotype and iron biomarkers data from participants recruited in Kenya, Uganda, Burkina Faso, South Africa and The Gambia, as well as from the Jackson Heart Study. These studies are described in detail in the Methods section.
Ethics oversight	Individual study site ethical approvals were obtained as detailed in the Methods.

Note that full information on the approval of the study protocol must also be provided in the manuscript.

Field-specific reporting

Please select the one below that is the best fit for your research. If you are not sure, read the appropriate sections before making your selection.

☒ Life sciences ☐ Behavioural & social sciences ☐ Ecological, evolutionary & environmental sciences

For a reference copy of the document with all sections, see nature.com/documents/nr-reporting-summary-flat.pdf

Life sciences study design

All studies must disclose on these points even when the disclosure is negative.

Sample size	The discovery cohort (n=3,928) had >80% power to detect genome-wide associations for variants with MAF ≥5% and effect size ≥0.33 SD. Severe malaria (7,957 cases; 7,746 controls) had >80% power for OR ≥1.15, and bacteraemia (1,970 cases; 4,013 controls) for OR ≥1.40.
Data exclusions	Individuals were excluded if they had identity by descent estimates ≥0.9 or had high SNP missingness rate. Genotypes were excluded if they had high missingness rate, sex discordance, or high heterozygosity metrics that indicate poor sample or genotyping quality.
Replication	The five discovery populations were independent enabling comparison between populations. We also replicated our findings in African Americans from the Jackson Heart Study.
Randomization	None for the primary results reported in the study.
Blinding	Not applicable.

Reporting for specific materials, systems and methods

We require information from authors about some types of materials, experimental systems and methods used in many studies. Here, indicate whether each material, system or method listed is relevant to your study. If you are not sure if a list item applies to your research, read the appropriate section before selecting a response.

Materials & experimental systems

n/a	Involved in the study	
☒	☐	Antibodies
☒	☐	Eukaryotic cell lines
☒	☐	Palaeontology and archaeology
☒	☐	Animals and other organisms
☒	☐	Clinical data
☒	☐	Dual use research of concern
☒	☐	Plants

Methods

n/a	Involved in the study	
☒	☐	ChIP-seq
☒	☐	Flow cytometry
☒	☐	MRI-based neuroimaging

Plants

Seed stocks

Report on the source of all seed stocks or other plant material used. If applicable, state the seed stock centre and catalogue number. If plant specimens were collected from the field, describe the collection location, date and sampling procedures.

Novel plant genotypes

Describe the methods by which all novel plant genotypes were produced. This includes those generated by transgenic approaches, gene editing, chemical/radiation-based mutagenesis and hybridization. For transgenic lines, describe the transformation method, the number of independent lines analyzed and the generation upon which experiments were performed. For gene-edited lines, describe the editor used, the endogenous sequence targeted for editing, the targeting guide RNA sequence (if applicable) and how the editor was applied.

Authentication

Describe any authentication procedures for each seed stock used or novel genotype generated. Describe any experiments used to assess the effect of a mutation and, where applicable, how potential secondary effects (e.g. second site T-DNA insertions, mosiacism, off-target gene editing) were examined.
