# Supplementary material for: African-specific genetic loci determine iron status and risk of severe malaria and bacteremia in African children
Source: Nat Commun. 2026 Apr 7;17:7068. doi: 10.1038/s41467-026-71567-w (PMC13392482; doi:10.1038/s41467-026-71567-w)
Supplement: Supplementary file 3 — Transparent Peer Review file [file 41467_2026_71567_MOESM3_ESM.pdf]

nature portfolio

Peer Review File

African-specific genetic loci determine iron status and risk of severe malaria and bacteremia in African children

Corresponding Author: Dr John Muriuki

This file contains all reviewer reports in order by version, followed by all author rebuttals in order by version.

Version 0:

Reviewer comments:

Reviewer #1

(Remarks to the Author)
The authors have investigated the genetic control of iron-related biomarkers in Africa. They performed genome wide association studies of transferrin, hepcidin, sTfR, ferritin, serum iron, and TSAT in discovery cohorts including people from Kenya, Uganda, Burkina Faso, South Africa and The Gambia. In addition, they used data from the Jackson Heart study as a replication cohort, and used MalariaGEN GWAS and Kenyan Bacteremia data for assessing the association of candidate SNPs with malaria and bacteremia, respectively. The hypothesis underlying this study was that iron homeostasis has an effect on bacterial and plasmodial infection.

The paper is clear and well written, and the methods are appropriate for the purpose of the study. The results are convincing and very interesting. In particular, the authors report several SNPs associated with iron-related biomarkers, such as transferrin levels, hepcidin levels, or soluble transferrin receptor levels. These associations were detected either in the grouped five African populations, or in specific populations. Among them, some SNPs were replicated in an independent cohort and previous GWAS in European populations. In addition, previously unreported loci (GTF3C5 and CHCHD7) were associated with transferrin levels or hepcidin levels. Moreover, GTF3C5 locus was associated with transferrin levels, severe malaria and bacteremia, while CHCHD7 was associated with hepcidin levels and bacteremia.

I have, nevertheless, some comments:
-the authors should state whether the identified SNPs were imputed SNPs (or genotyped) in Table 1. If it is the case, they could state the quality of imputation. In addition, they should state this limitation in the discussion.
- the authors should state relevant functional annotation for each SNP within Table 1. They should state in methods whether they used positional, eQTL, or 3D chromatin interaction mapping for mapping for identifying genes using FUMA.
-the authors should provide a supplementary Table that provide the quality of imputation and the functional annotation for each SNP associated with transferrin levels, hepcidin levels, or soluble transferrin levels (see Figures 2, 3, 4, and 5).

Reviewer #2

(Remarks to the Author)
Muriuki et al. conducted a series of GWAS for iron-related biomarkers using five African samples and identified some novel associations. The authors also provide insights into the reasons behind the newly detected associations. GWAS for underrepresented populations is crucial for further biological research and the realization of equitable precision medicine. I have some comments on this paper from a scientific perspective.

GTF3C5 locus for transferrin levels was detected in East Africa (Kenya and Uganda) through a meta-analysis, but not in other regions. This variant is also common in European populations, yet it hasn't been reported in studies of European populations. To explain this discrepancy, the authors analyzed haplotype configurations. They found that Haplotype 2 is common in Kenya, Uganda, and South Africa, but absent in The Gambia and Burkina Faso. However, based on S Fig 4, I believe Haplotype 2 is present in Burkina Faso. The haplotype configuration is unclear, so please provide the data as a supplementary material. Since the European population is also discussed, it would be beneficial to analyze haplotype configurations in European populations as well. I believe the 1000 Genomes data is sufficient for this purpose. To support

the authors' hypothesis, I believe a haplotype-level association test is necessary to demonstrate the (negative) risk effect observed only by Haplotype 2, not Haplotype 1, even when the rs290504-T allele is present.

Another comment for GTF3C5 is that I suggest the authors conduct statistical fine mapping in East African populations first. SNPs with high PIP, excluding rs2905094, could potentially explain the discrepancy in the association between regions. This possibility has not been explored.

The authors identified the novel CHCHD7-SDR16C5 locus for hepcidin and noted that "This region exhibits evidence of positive selection." Examining Figure 4, the core region of the haplotype involving rs73596248 spans from 57.13 to 57.30 Mb on chromosome 8. However, the reported selected region from PopHumanScan ranges from 56,967,474 to 57,015,445 (hg19), which does not align with the locuszoom plot presented in Figure 4 at a glance. In my opinion, the authors should provide additional explanations to establish a direct connection between the associated variant and the selected region.

The authors focused solely on the association of SMARCA5, which was only detected in a single Kenyan GWAS. As shown in S Fig1, there would also be other solitary significant associations, such as transferrin in Kenya and South Africa, ferritin in Uganda, and sTfR in The Gambia. Why did the authors ignore these associations and only focus on SMARCA5?

Minor points:
- The authors used “inflammation” as one of the traits; rather, would it be better to use the term “chronic inflammation”?
- It's written in STab1 that participants in the Jackson Heart Study weren't exposed to malaria, but the table shows "n/a" for this study. Should it be 0, or if the ascertainment wasn't enough, it shouldn't be described as "not exposed to malaria"?
- This reviewer believes the number of participants who failed the sex check, which is 58 (4.4%), in STab2 for Kenya, is too high. Is there any effort being made to prevent sample swapping?
- Samples from Kenya and The Gambia should be genotyped using the same H3Africa array. However, only The Gambia's results showed a significantly higher number of duplicated variants (182,365) compared to Kenya's (28) in STab 2. This discrepancy raises questions about the reasons behind it.

Reviewer #3

(Remarks to the Author)
Thank you for asking me to review this manuscript. I agree with the authors’ view of the importance of topic and welcome their efforts to contribute to our understanding in an understudied population.

Key results and significance
The key message that I got from the paper is that the genetic determinants of iron status in African populations are different from European populations and that this likely reflects the greater genetic diversity of the population and selective pressure exerted by malaria and other infections. This I believe was the main aim of the paper. I was less clear about the clinical and public health significance of the results. The stated new findings were the identification of a new loci for transferrin in GTF3C5 responsible for conferring protection against severe malaria and bacteriamia; a new loci for hepcidin in CHCHD7/SDR16C5, and variant rs73596248 associated with an increase hepcidin levels that conferring protected against bacteremia. However in the results section it is clearer that there is only an association with the risk of klebsiella and staph aureus bacteriaemia, but not with bacteraemia overall or with severe malaria. They also found that the rare Dantu blood group was associated with soluble transferrin receptor levels, (and confirmed previous reports that this blood group is associated with protection against severe malaria and bacteraemia). Interestingly the authors quote their own study (9) which shows link between iron status and malaria but which also did not show any relationship with hepcidin.

Aims and study design
Tt seems that there were two main aims: Firstly “To enhance discovery of genetic determinants of iron status in an understudied population (line 106) and secondly “To understand the relationship between iron and infection, we examined the effect of these variants on risk of severe malaria and bacterial infections” (line 109).

It felt to me that there was a much stronger rationale for the chosen study design for the first aim, and this was also reflected in the detail in which the findings were presented, and the robustness of the findings:

For the second aim, the presentation of results was not very clear, the interpretation of results less convincing and some of the details re. methods and statical analysis were missing.

Data and methodology
As mentioned above, I think the methods for Discovery and Replication GWAS seem appropriate although I have some concerns about the how the heterogeneity of contributing study designs and I and the resulting differences and gaps in the data collected. For example that transferrin was not measured in two sites and why α1-Antichymotrypsin being used as a marker of inflammation in one of the studies, rather than a more standard marker such as CRP. I also think it would have been useful to show red cell indices, other than haemaglobin, as more clinically relevant reflection of iron status than just the biochemical markers presented.
For the study looking for an association between the SNPs and severe malaria and bacteraemia I there was less description of the methods and the data making it difficult to comment on. Providing more information including a descriptive table of the population characteristics would be helpful. I was specifically interested to understand when these samples had been taken given the changing malaria epidemiology and had to go to other publications to find this. I was surprised to learn that that the

original Malariagen study was conducted in Kilifi (conducted 1995-2008). Indeed a recently published study by the group designed specifically to explore the relationship between the Dantu and bacteriaemia, analysed separately data from 1998-2003, a period of high transmission and data from 2003-2010, a period of low transmission. They reported that the protective effect of Dantu against all-cause bacteremia was only significant during the high malaria transmission period suggesting that the protective effect was most likely mediated indirectly by its protective effect against malaria. It is not clear why a similar approach was not taken in this current study and somewhat contradicts which the authors state "….the Dantu NE blood group phenotype, is under strong selective pressure for protection against malaria along the Kenyan coast." (Line 255).

Analytical approach
In the section of statistical analysis section (line 764-814), there is no description of how the data were analysed for the association between SNPs and bacteriaemia.
In the section on sample size and power (line 840-846), there is no description of the sample size and power calculations for the studies looking for association of SNPs with severe malaria and bacteriemia.

Clarity and context
I this paper would benefit from being written more clearly, coherently and concisely. This would make it both stronger and more accessible. There are important details missing and very limited mention of any study limitations which I think are essential to address. I think it would also be helpful to clarify what's new and significant in terms of implications for clinical practice or public health. Perhaps one way to make this clearer would be to give an example of how results of GWAS studies of iron status in other (eg European populations) have impacted on clinical care or outcomes?

Version 1:

Reviewer comments:

Reviewer #2

(Remarks to the Author)
I appreciate the authors' efforts to address my comments. However, I still have some concerns.

I appreciate the authors included the statement that the frequency of rs2905094-T was 9 % in Burkina Faso. Nonetheless, they refer to this as a "low frequency." Generally, "low frequency" denotes frequencies of 5 % or less, often even below 1 %. A 9 % frequency would be considered common rather than low.

The authors claim that they cannot publish the haplotype configuration for ethical reasons. I am not requesting individual-level data; it would be sufficient to provide a representative configuration and its count. The data in SFig 4 are too vague. For example, the manuscript states that Hap1 is present at less than 20 % in South Africa and Hap2 at about 15 %. Yet, looking at the third row from the top in SFig 4, Hap1 appears to be more than twice as frequent as Hap2, which is inconsistent with the manuscript. Displaying only the representative SNP alleles that define each haplotype and presenting them in a table with numeric values would resolve this. What matters most is identifying which haplotype carries the causal variant. Differences in haplotype configuration between EUR and other populations would also be clearer if shown using the representative alleles and their counts.

From the haplotype association results, the hypothesis that the association observed in these populations is driven by Hap2 is not supported.

Summarizing the findings thus far: rs2905094 is significantly associated with transferrin levels only in East African populations comprising Kenya and Uganda. In the fine-mapping of this group, rs2905094 has the highest PIP of about 0.57. The rs2905094-T allele is common in East Africa, Other Africa, and Europe. The data do not support the notion that a causal variant lies on a specific haplotype present only in East Africans and absent in other groups.

The explanation for the lack of association with rs2905094 outside East Africa remains insufficient. We cannot rule out issues related to quality control or population structure, and the absence of association in other groups suggests that the signal was not replicated. The tone regarding the association of this SNP rs2905094 should be moderated.

Reviewer #3

(Remarks to the Author)
Thank you for asking me to review the revised manuscript.
I am happy with how the authors addressed our concerns and believe that it has resulted in a much stronger and clearer manuscript.

Open Access This Peer Review File is licensed under a Creative Commons Attribution 4.0 International License, which permits use, sharing, adaptation, distribution and reproduction in any medium or format, as long as you give appropriate credit to the original author(s) and the source, provide a link to the Creative Commons license, and indicate if changes were made.
In cases where reviewers are anonymous, credit should be given to 'Anonymous Referee' and the source.
The images or other third party material in this Peer Review File are included in the article's Creative Commons license, unless indicated otherwise in a credit line to the material. If material is not included in the article's Creative Commons license and your intended use is not permitted by statutory regulation or exceeds the permitted use, you will need to obtain permission directly from the copyright holder.
To view a copy of this license, visit https://creativecommons.org/licenses/by/4.0/

REVIEWER COMMENTS

Reviewer #1 (Remarks to the Author):
The authors have investigated the genetic control of iron-related biomarkers in Africa. They performed genome wide association studies of transferrin, hepcidin, sTfR, ferritin, serum iron, and TSAT in discovery cohorts including people from Kenya, Uganda, Burkina Faso, South Africa and The Gambia. In addition, they used data from the Jackson Heart study as a replication cohort, and used MalariaGEN GWAS and Kenyan Bacteremia data for assessing the association of candidate SNPs with malaria and bacteremia, respectively. The hypothesis underlying this study was that iron homeostasis has an effect on bacterial and plasmodial infection.

The paper is clear and well written, and the methods are appropriate for the purpose of the study. The results are convincing and very interesting. In particular, the authors report several SNPs associated with iron-related biomarkers, such as transferrin levels, hepcidin levels, or soluble transferrin receptor levels. These associations were detected either in the grouped five African populations, or in specific populations. Among them, some SNPs were replicated in an independent cohort and previous GWAS in European populations. In addition, previously unreported loci (*GTF3C5* and *CHCHD7*) were associated with transferrin levels or hepcidin levels. Moreover, *GTF3C5* locus was associated with transferrin levels, severe malaria and bacteremia, while *CHCHD7* was associated with hepcidin levels and bacteremia.

Response: We thank the reviewer for this positive feedback, and for recognizing the clarity, methodological rigor, and significance of our study.

I have, nevertheless, some comments:
-the authors should state whether the identified SNPs were imputed SNPs (or genotyped) in Table 1. If it is the case, they could state the quality of imputation. In addition, they should state this limitation in the discussion.

Response: We appreciate the reviewer's point that highlighting associations with poorly imputed SNPs could lead to erroneous conclusions. To address this concern, we have added Supplementary Table 4, which includes the source of each of the lead SNPs (whether genotyped or imputed) and the INFO score, where relevant. We have updated Table 1 to indicate which lead SNPs were directly genotyped and which were imputed. All of the lead SNPs that we are presenting are either genotyped or of high quality (INFO >0.95), and we have added a statement to this effect in the Results section, as follows:

Page 3: "All lead SNPs were either genotyped or imputed with high quality (INFO>0.95; Supplementary Table 4)."

Additionally, we have revised the Discussion to acknowledge this as a limitation as follows:

Page 10: "Additionally, some lead SNPs were imputed, although imputation quality was high (INFO >0.95), and variants with small effect sizes may not have been detectable with the current sample size. Larger genomic studies within Africa are needed to detect smaller-effect variants and refine genetic architecture across populations."

- the authors should state relevant functional annotation for each SNP within Table 1. They should state in methods whether they used positional, eQTL, or 3D chromatin interaction mapping for mapping for identifying genes using FUMA.

Response: We have updated Table 1 to include the functional annotation of each lead SNP. We have also revised the Methods section to clarify how we conducted functional annotation as follows:

Pages 23-24: "We performed functional annotation of lead GWAS variants using the Ensembl Variant Effect Predictor (VEP, GRCh37, https://www.ensembl.org/Tools/VEP), reporting the most severe consequence per variant based on canonical transcripts. To identify candidate target genes, we used the FUMA GWAS platform (https://fuma.ctglab.nl/), applying three complementary mapping strategies: (1) positional mapping within ±10 kb of gene boundaries, (2) cis-eQTL mapping using data from GTEx v8 across multiple tissues, and (3) chromatin interaction mapping based on Hi-C datasets from relevant cell types. In parallel, we queried the African Functional Genomics Resource (https://github.com/smontgomlab/AFGR) to identify eQTLs in African ancestry populations. Genes implicated by any of these approaches were considered candidate target genes."

-the authors should provide a supplementary Table that provide the quality of imputation and the functional annotation for each SNP associated with transferrin levels, hepcidin levels, or soluble transferrin levels (see Figures 2, 3, 4, and 5).

Response: We have added functional annotations, as determined by the Ensembl Variant Effect Predictor (VEP), to Supplementary Tables 6, 7, 10, and 11 that report SNPs associated with transferrin at the *TF* locus, transferrin at the *GTF3C5* locus, hepcidin, and soluble transferrin receptor (sTfR) levels. These annotations include the predicted consequence and genomic context of each variant. Additionally, we have expanded Supplementary Table 4 to include SNP annotations and imputation INFO scores for all genome-wide significant SNPs presented in Figures 2–5.

Reviewer #2 (Remarks to the Author):
Muriuki et al. conducted a series of GWAS for iron-related biomarkers using five African samples and identified some novel associations. The authors also provide insights into the reasons behind the newly detected associations. GWAS for underrepresented populations is crucial for further biological research and the realization of equitable precision medicine. I have some comments on this paper from a scientific perspective.

Response: We thank the reviewer for noting the importance of GWAS in underrepresented populations.

GTF3C5 locus for transferrin levels was detected in East Africa (Kenya and Uganda) through a meta-analysis, but not in other regions. This variant is also common in European populations, yet it hasn't been reported in studies of European populations. To explain this discrepancy, the authors analyzed haplotype configurations. They found that Haplotype 2 is common in Kenya, Uganda, and South Africa, but absent in The Gambia and Burkina Faso.

However, based on S Fig 4, I believe Haplotype 2 is present in Burkina Faso. The haplotype configuration is unclear, so please provide the data as a supplementary material. Since the European population is also discussed, it would be beneficial to analyze haplotype configurations in European populations as well. I believe the 1000 Genomes data is sufficient for this purpose. To support the authors' hypothesis, I believe a haplotype-level association test is necessary to demonstrate the (negative) risk effect observed only by Haplotype 2, not Haplotype 1, even when the rs290504-T allele is present.

Response: We thank the reviewer for these insightful comments, which have improved the clarity and depth of our analyses. We re-examined the *GTF3C5* haplotype structure and confirmed that Haplotype 2 is indeed present, although at a low frequency (9%) in Burkina Faso. We have made this correction in the Results section as follows:

Page 4: "Haplotype 2 accounted for 40%, 25% and 15% of chromosomes from Kenya, Uganda, and South Africa, respectively, but was absent in The Gambia and at low frequency in Burkina Faso (9%)."

While we generated full haplotype configuration data for all study populations, we are unable to include these in the Supplementary Data. Our more recent studies are conducted with open access to sequence data in mind, but these data come from older studies with stricter ethical and data governance restrictions that prohibit sharing individual-level genomic information. However, we have shared the detailed haplotype configurations with the journal in this resubmission to allow sharing in confidence with the reviewer to enable verification of our results, on the understanding that the dataset is not for publication.

Given that rs2905094 is common in European ancestry populations, we also analyzed the *GTF3C5* haplotype configuration using 1000G EUR data as suggested. However, due to differences in SNP coverage, the European haplotype block had only 110 SNPs (versus 209 in African datasets), precluding direct alignment with African Haplotype 1 and 2. Instead, we identified two major EUR haplotypes (Haplotype 3 and Haplotype 4). Based on shared SNP patterns within overlapping regions, Haplotype 3 shows partial similarity to African Haplotype 2, while Haplotype 4 resembles Haplotype 1. It is therefore possible that although rs2905094-T is common in Europeans, it resides on a distinct haplotype, which may explain the lack of association in European iron GWAS. We have included the EUR haplotype results in Supplementary Figure 6 and in the Results section as follows:

Page 5: "Although rs2905094 is more common in Europeans, analysis of 1000 Genomes EUR data revealed two predominant haplotypes (Haplotype 3 and Haplotype 4; Supplementary Fig. 6) that could differ from African haplotypes, suggesting that the rs2905094-T allele may segregate on a different haplotype background in Europeans, potentially explaining the absence of an association in European iron GWAS."

We have also updated the Methods haplotype analyses section as follows:

Page 24: "Since rs2905094 is common in European ancestry populations, we also analyzed the GTF3C5 haplotype configuration using 1000 Genomes Project EUR data."

To test whether the association signal was driven by a specific haplotype carrying the derived rs2905094-T allele, we performed haplotype-level association tests for transferrin levels in East African participants, adjusting for age, sex, country, and ten genetic principal components. Both Haplotype 1 and Haplotype 2 were associated with lower transferrin levels. However, conditioning on rs2905094 genotype abolished both associations ($P > 0.4$), and among carriers of the rs2905094-T allele, neither haplotype showed additional effects suggesting that the East-African signal may be driven by rs2905094 or an unidentified variant in strong linkage disequilibrium with it, rather than by an independent haplotype variant. We have added these analyses to the Methods section as follows:

Page 24: "To determine whether the association signal at GTF3C5 was driven by a specific haplotype carrying the derived rs2905094-T allele, we performed haplotype-level association analyses for transferrin levels among East African participants. Haplotype dosages (0/1/2) were estimated from phased genotype data and incorporated into linear regression models of inverse-normalized transferrin levels, adjusting for age, sex, study site, and the first ten principal components of ancestry."

We have also included the findings in Supplementary Table 8 and in the Results and Discussion sections as follows:

Pages 4-5: "Through haplotype-level analyses in East Africans, both Haplotypes 1 and 2 were associated with lower transferrin levels, but these effects disappeared after conditioning on rs2905094, indicating that the signal is driven by rs2905094 or an unidentified variant in strong LD with rs2905094 (Supplementary Table 8)."

Pages 7-8, Discussion: "However, haplotype-level association analysis did not provide evidence for a haplotype-specific effect, suggesting the association is more likely to be driven by rs2905094 itself or a variant in strong LD with rs2905094."

Another comment for *GTF3C5* is that I suggest the authors conduct statistical fine mapping in East African populations first. SNPs with high PIP, excluding rs2905094, could potentially explain the discrepancy in the association between regions. This possibility has not been explored.

Response: Thank you for this insightful suggestion. We have now conducted statistical fine-mapping using the SuSiE-R package based on meta-analyzed summary statistics from Kenya and Uganda. These analyses identified a single credible set centered on rs2905094, which had the highest posterior inclusion probability (PIP = 0.57), followed by rs2905095 (PIP = 0.28) and rs2905089 (PIP = 0.08). All remaining variants had PIP < 0.03, suggesting a single likely causal signal at *GTF3C5*. We have updated the Methods section as follows:

Page 23: "To identify variants driving the East African *GTF3C5* locus, we performed statistical fine-mapping using the *susieR* package[68] in R version 4.2.3 based on meta-analyzed transferrin GWAS summary statistics from East African cohorts (Kenya and Uganda). A region-specific LD matrix, derived from the merged East African genotype data, was used to accurately model local LD structure. We specified up to 10 single-effect components (L = 10) to allow for multiple independent causal signals within the

locus. Posterior inclusion probabilities (PIPs) were estimated for each variant, and 95% credible sets were defined to identify variants most likely to drive the association signal.

We have also added these fine-mapping results to Supplementary Fig. 5 and updated the Results and Discussion sections as follows:

Page 5, Results: "Through fine-mapping, we identified a single credible set centered on rs2905094 which had the highest posterior inclusion probability (PIP = 0.57), with rs2905095 and rs2905089 contributing minor probabilities (Supplementary Fig. 5), consistent with a single causal signal at *GTF3C5*."

Page 8, Discussion: "Consistent with this, fine mapping analyses also found most evidence for an effect of rs2905094 and little evidence for multiple independent associations in this region. Although these analyses do not fully explain the population-specific nature of the signal, they emphasize the importance of examining regional genomic diversity, particularly in African populations, and there remains the possibility that divergent LD patterns and/or gene–environment interactions may shape the underlying genetic effects at this locus.[19,23]"

The authors identified the novel *CHCHD7-SDR16C5* locus for hepcidin and noted that "This region exhibits evidence of positive selection." Examining Figure 4, the core region of the haplotype involving rs73596248 spans from 57.13 to 57.30 Mb on chromosome 8. However, the reported selected region from PopHumanScan ranges from 56,967,474 to 57,015,445 (hg19), which does not align with the locuszoom plot presented in Figure 4 at a glance. In my opinion, the authors should provide additional explanations to establish a direct connection between the associated variant and the selected region.

Response: We agree that the initially cited PopHumanScan region (56.97–57.02 Mb) does not perfectly overlap with the hepcidin-associated LD block shown in Figure 4. Upon inspection, we found that PopHumanScan contains no entries covering the narrower window (57.13–57.30 Mb), which explains the discrepancy. To directly assess evidence of selection at this locus, we re-computed the integrated haplotype score (iHS) using selscan (v2.1.0). While examining the region suggested by the reviewer (56.97–57.02 Mb), we observed that the signal of association and extended haplotype homozygosity actually begins around 57.05 Mb. We therefore expanded the window to chr8:57.05–57.30 Mb to capture the full extent of local linkage disequilibrium, as illustrated in Figure 4.

We computed iHS across all five African cohorts, and the results (now included in Supplementary Data 1) consistently show elevated absolute iHS values with a shift toward negative scores (Supplementary Fig. 8), indicative of recent positive selection acting on the derived allele at this locus. We have now updated the Methods as follows:

Page 24: "Selection scan using integrated haplotype scores
Since the LD pattern around the *CHCHD7/SDR16C5* locus on chromosome 8 was suggestive of an extended haplotype, we investigated extended haplotype homozygosity (EHH) patterns for each of the five African sites. Variants were restricted to chromosome 8 positions 57,050,000–57,300,000 bp (GRCh37/hg19 coordinates) to encompass the LD block surrounding the lead hepcidin GWAS variant (rs73596248). SNP positions within the target region were used to interpolate recombination distances

from the 1000 Genomes Project Phase 3 genetic map. Integrated haplotype scores (iHS) were computed using selscan v2.1.0.[69] SNPs with MAF ≥0.01 were included. The iHS statistic was calculated for variants in the target region, measuring the decay of EHH for ancestral and derived alleles. Using selscan's normalization procedure, raw iHS were standardized by allele frequency bins (n=10) across the genome to account for variation in allele frequencies. Haplotype signals were visualized using *ggplot2* package in R v4.3.2, with loess smoothing applied to highlight local trends in normalized iHS values across the genomic region.

We have also updated the Results and Discussion sections as follows:

Page 5, Results: "The regional plot suggests that this association is within an extended haplotype (Fig. 4a) and we observed elevated absolute integrated haplotype score (iHS) values across all African cohorts (Fig. 4b and Supplementary Data 1), indicative of extended haplotype homozygosity. Genome-wide loess-smoothed profiles revealed a consistent trend toward negative iHS values suggesting recent selection acting on the derived allele (Supplementary Fig. 8)."

Page 8, Discussion: "we observed elevated absolute iHS values, with a shift toward negative values, suggesting recent positive selection acting on the derived allele at the locus probably to protect against pathogens."

The authors focused solely on the association of *SMARCA5*, which was only detected in a single Kenyan GWAS. As shown in S Fig1, there would also be other solitary significant associations, such as transferrin in Kenya and South Africa, ferritin in Uganda, and sTfR in The Gambia. Why did the authors ignore these associations and only focus on *SMARCA5*?

Response: We appreciate the reviewer's careful attention to the country-specific GWAS results. As noted, several solitary genome-wide significant associations appear in Supplementary Figure 1, including for transferrin, ferritin, and sTfR in individual country-level analyses. We had examined these signals closely and found that they were driven by rare variants (MAF <2%), which limits statistical power for replication and increases the likelihood of false positives.

In contrast, we chose to highlight the Kenyan-specific *SMARCA5* / Dantu locus because the associated variants were relatively common (~9% MAF) in this population and imputed with high quality. Importantly, the association lies within the well-known Dantu region, which is under strong positive selection along the Kenyan coast due to its known protective effect against severe malaria, further supporting the biological plausibility of this finding.

To clarify this rationale, we have added a note to the Supplementary Figure 1 legend stating that "Several genome-wide significant associations observed in individual country-level analyses (for example, sTfR in The Gambia) were driven by rare variants (MAF < 2%). Due to their low frequency and limited power for replication, these signals were not explored further in the current study."

Minor points:

- The authors used "inflammation" as one of the traits; rather, would it be better to use the term "chronic inflammation"?

Response: In our study, "inflammation" was defined based on elevated acute-phase reactants, specifically C-reactive protein (CRP >5 mg/L) or α1-antichymotrypsin (ACT >0.6 g/L), consistent with established guidelines for identifying inflammation in children. Since CRP and ACT were measured cross-sectionally at one timepoint (together with markers of iron status), we cannot be certain whether inflammation has persisted over time as 'chronic inflammation' or if it is acute inflammation. We therefore believe that "inflammation" is the more accurate term. We have clarified this definition in the Methods as follows:

Page 21: "Since iron biomarkers can be affected by inflammation or infection,[1] and it is important to account for inflammation when defining iron deficiency, we defined inflammation as CRP >5 mg/L or ACT >0.6 g/L (in Gambian children), consistent with established guidelines for identifying inflammation in children.[59]

- It's written in STab1 that participants in the Jackson Heart Study weren't exposed to malaria, but the table shows "n/a" for this study. Should it be 0, or if the ascertainment wasn't enough, it shouldn't be described as "not exposed to malaria"?

Response: We agree that "n/a" may be ambiguous in this context. As the Jackson Heart Study was conducted in a non-endemic region with no known exposure to malaria, we have updated Supplementary Table 1 to "report 0/2868 under the n/total column and 0.0% under the prevalence column for malaria parasitemia, consistent with how we reported data for South Africa"

- This reviewer believes the number of participants who failed the sex check, which is 58 (4.4%), in STab2 for Kenya, is too high. Is there any effort being made to prevent sample swapping?

Response: We acknowledge that the sex mismatch rate in the Kenyan cohort (58 of 1,312 samples; 4.4%) is higher than typically expected. This cohort was part of a previously conducted longitudinal study of malaria immunity in children in Kilifi, Kenya. Blood samples were collected during annual cross-sectional surveys and archived at -80°C. For this study, samples were selected retrospectively from archived plasma based on availability, which may have introduced a higher risk of mislabeling or mismatch across visits. While this might have contributed to the elevated sex check failure rate, we applied rigorous genotype quality control procedures and excluded all affected samples from downstream analyses.

- Samples from Kenya and The Gambia should be genotyped using the same H3Africa array. However, only The Gambia's results showed a significantly higher number of duplicated variants (182,365) compared to Kenya's (28) in STab 2. This discrepancy raises questions about the reasons behind it.

Response: We confirm that there was an error in reporting the number of individuals and variants at each QC step for the Gambian population. Initially, we reported that 2,297,939 variants were typed and mapped to Build 37 in the Gambian dataset. Upon investigation, we realized that these figures were based on an earlier version of the Gambian data, in which the 528- and 46-sample batches were merged prior to quality control. This pre-QC merge resulted in an inflated variant count due to multiple probes

mapping to the same genomic positions on the H3Africa genotyping array. Some entries contained placeholder alleles (e.g., allele coded as "0"), introduced during genotype calling. For example, in the 528-sample file, a variant might appear as 1:821501:0:T, while in the 46-sample file, the same position appears as 1:821501:C:T. These mismatches led to a large number of apparent duplicates during the QC process. Importantly, this pre-QC merged file was not used for imputation or downstream analyses. Instead, we had merged the 528 and 46 files after QC, ensuring that only clean, non-duplicated variants were included in the final dataset.

We have now updated Supplementary Table 2 to reflect the correct number of individuals and variants at each QC step for the Gambian population. We confirm that this correction does not affect any downstream analyses or study conclusions. We have also verified that the QC steps reported for the other populations are accurate.

Reviewer #3 (Remarks to the Author):
Thank you for asking me to review this manuscript. I agree with the authors' view of the importance of topic and welcome their efforts to contribute to our understanding in an understudied population.

Key results and significance
The key message that I got from the paper is that the genetic determinants of iron status in African populations are different from European populations and that this likely reflects the greater genetic diversity of the population and selective pressure exerted by malaria and other infections. This I believe was the main aim of the paper. I was less clear about the clinical and public health significance of the results.

Response: We thank the reviewer for confirming the importance of the topic and the importance of studying under-represented populations. We agree that the central contribution of our study is the identification of African-specific genetic determinants of iron status and their contrast with European populations, likely reflecting both the greater genetic diversity in Africa and the selective pressures of infectious diseases such as malaria and bacteremia. Our study additionally highlights that genetic risk scores and instrumental variables for Mendelian randomization (MR) studies identified from European populations are unlikely to be useful in African populations, thus underlining the need for genomic studies in continental Africa to address important clinical questions that could be answered through MR studies. Our findings have potential public health relevance especially for iron supplementation programs since genetic differences in iron regulation may modify the balance between correcting deficiency and increasing infection risk. To better highlight these clinical and public health implications while acknowledging that further work is needed, we have revised the Discussion as follows:

Page 10: "These findings have important implications for understanding genetic and environmental interactions shaping iron regulation and infection risk in African populations. While large European iron-GWAS have been foundational for several Mendelian randomization studies that clarified causal relationships relevant to clinical guidelines,[45,46] our results highlight the need for Africa-centric genomic data since genetic risk scores and instrumental variables identified in European populations were

poorly predictive in African populations. African-specific data is needed to guide diagnostic screening and inform targeted interventions and equitable global health strategies. Our findings have potential public health relevance for iron supplementation programs since genetic differences in iron regulation may modify the balance between correcting deficiency and increasing infection risk, underscoring the importance of population-specific approaches. For instance, routine or universal iron supplementation or fortification, especially in the context of elevated hepcidin, may lead to iron accumulation in the gut or dysregulated iron distribution, potentially increasing susceptibility to enteric and systemic infections.[8,47] Future studies should explore the mechanistic pathways linking the identified variants to iron-pathogen interactions to inform safe and targeted interventions in African settings."

The stated new findings were the identification of a new loci for transferrin in *GTF3C5* responsible for conferring protection against severe malaria and bacteriamia; a new loci for hepcidin in *CHCHD7/SDR16C5*, and variant rs73596248 associated with an increase hepcidin levels that conferring protected against bacteremia. However in the results section it is clearer that there is only an association with the risk of klebsiella and staph aureus bacteriaemia, but not with bacteraemia overall or with severe malaria.

Response: We agree that our initial wording may have overstated the breadth of protection associated with the hepcidin locus. As the reviewer correctly points out, the variant rs73596248 at the *CHCHD7/SDR16C5* locus was associated with increased hepcidin levels and reduced risk of *Klebsiella pneumoniae* and *Staphylococcus aureus* bacteremia, but not with overall bacteremia or with severe malaria. We have adjusted the wording in the Abstract and ensured consistency in Results, Discussion and Conclusion to avoid implying broader protection than was supported by the data.

They also found that the rare Dantu blood group was associated with soluble transferrin receptor levels, (and confirmed previous reports that this blood group is associated with protection against severe malaria and bacteraemia). Interestingly the authors quote their own study (9) which shows link between iron status and malaria but which also did not show any relationship with hepcidin.

Response: We agree that our previous study (https://doi.org/10.1093/cid/ciy791) demonstrated a relationship between iron status and malaria risk but did not identify an association with hepcidin. In the current study, we do not report a direct association between Dantu and hepcidin levels. Rather, we observed that the Dantu blood group locus was associated with soluble transferrin receptor (sTfR) levels, which we highlighted in the context of Dantu's well-established protective effect against severe malaria and bacteremia. If the reviewer's comment refers to sTfR, we agree that in our previous study we did not find any relationship between sTfR levels and malaria and have now made this clear in the Discussion as follows:

Page 9: "Although sTfR levels may not be directly linked to malaria infection in observational analyses,[9] elevated sTfR reflects expanded erythropoietic activity and can indicate ID, which is itself associated with protection against malaria infection.[9]"

If the reviewer's comment instead refers to the *CHCHD7/SDR16C5* locus, we note that this variant was associated with increased hepcidin levels and reduced risk of *Klebsiella pneumoniae* and *Staphylococcus aureus* bacteremia. However, it was not associated with

severe malaria, consistent with our earlier mentioned study (https://doi.org/10.1093/cid/ciy791) that likewise found no evidence for a link between hepcidin and malaria risk. We have clarified this point in the Discussion as follows:

Page 8: "However, we found no association with severe malaria, consistent with our previous prospective study that found no relationship between hepcidin concentrations and malaria risk.[9]"

Aims and study design
Tt seems that there were two main aims: Firstly "To enhance discovery of genetic determinants of iron status in an understudied population (line 106) and secondly "To understand the relationship between iron and infection, we examined the effect of these variants on risk of severe malaria and bacterial infections" (line 109).

It felt to me that there was a much stronger rationale for the chosen study design for the first aim, and this was also reflected in the detail in which the findings were presented, and the robustness of the findings:

For the second aim, the presentation of results was not very clear, the interpretation of results less convincing and some of the details re. methods and statical analysis were missing.

Response: We thank the reviewer for acknowledging the robustness of our analyses and findings from the iron GWAS, which formed the first aim of our study. To strengthen the presentation of the second aim, examining the effect of identified iron-related variants on severe malaria and bacteremia, we have substantially revised the manuscript. Specifically, we have expanded the Methods section to include detailed descriptions of the infection GWAS datasets, analytical approaches, and newly added power calculations for these follow-up analyses as follows:

Pages 20 and 21:
***MalariaGEN consortium*: This was a GWAS study of severe malaria in ~17,000 severe malaria cases and population controls from 11 countries in Africa (Kenya, Malawi, Tanzania, The Gambia, Mali, Burkina Faso, Ghana, Nigeria, Cameroon), Asia (Vietnam), and Oceania (Papua New Guinea) as previously described.[24] Sample collection, processing, genotyping, quality control, imputation, GWAS, and population characteristics are described in details elsewhere.[24,57] Severe malaria was defined according to WHO criteria[58] and included diagnoses of severe malaria anemia, cerebral malaria, and other malaria-related symptoms. Association testing for each variant and severe malaria was performed using SNPTEST software in each study population adjusting for principal components under additive, dominant, recessive, heterozygote and general models of association. In the current study, we extracted additive model estimates for African populations. For the East-Africa specific *GTF3C5* lead SNP, rs2905094, we retrieved population-specific results from all of the nine African populations in the MalariaGEN database and applied fixed-effects meta-analyses by East (Kenya, Malawi, and Tanzania) and West (The Gambia, Mali, Burkina Faso, Ghana, Nigeria, and Cameroon) African sites. For the association between the hepcidin lead SNP, rs73596248 and severe malaria, we meta-analyzed estimates from all of the nine African populations in the MalariaGEN database. Since the sTfR GWAS signal is specific to Kenyan populations, we used data from the MalariaGEN Kenyan GWAS to**

determine the association between the top sTfR GWAS SNPs and severe malaria. The lead SNP, rs552439837, was not present in the severe malaria case–control dataset; therefore, we used estimates for rs141274959, a genome-wide significant sTfR GWAS variant in strong LD with rs552439837 ($r^2 = 0.9$).

***The Kenyan Bacteremia GWAS*: This GWAS included 1,970 Kenyan children (<13 years old) admitted to Kilifi County Hospital between 1998 and 2010 with culture-confirmed community-acquired bacteremia, and 4,013 healthy community controls, as previously described.[25] Controls were infants aged 3-12 months recruited between 2006 and 2008 from the same geographic area through the Kilifi Genetic Cohort Study. All participants were residents of Kilifi County on the coast of Kenya. Genotyping was performed in two phases: the discovery phase employed the Affymetrix SNP 6.0 array (787,861 SNPs) in 1,536 cases and 2,677 controls, while the replication phase used the Illumina ImmunoChip (143,100 SNPs) in an additional 434 cases and 1,336 controls, yielding a total of 1,970 bacteremia cases and 4,013 controls. Whole-genome imputation was carried out using SHAPEIT and IMPUTE2 with the 1000 Genomes Phase 3 reference panel. Demographic data for cases and controls and distribution of the most common bacterial species in the discovery and replication sets are described elsewhere.[25] In the current study, associations between conditionally independent iron-related SNPs and bacteremia were assessed using additive logistic regression models, adjusted for age, sex, and principal components. Analyses were performed for both overall bacteremia and stratified by the most common bacterial species.**
Page 23: "For SNP-bacteremia associations, we identified our African-specific SNPs in the largest GWAS of bacteremia in African populations,[25] merged the genotypes with the hospital clinical data, and conducted logistic regression analyses adjusting for age, sex, and principal components."

Page 24: "For the association between lead SNPs and severe malaria or bacteraemia, we estimated that the severe malaria dataset (7,957 cases, 7,746 controls) had ~80% power to detect an odds ratio ≥1.15 for a variant with MAF = 5% at $\alpha = 0.05$, while the bacteremia dataset (1,970 cases, 4013 controls) had ~80% power to detect an odds ratio ≥1.40."

We have also added Supplementary Table 9, which summarizes the characteristics of the infection GWAS populations by identified African-specific iron-related lead variants, to improve clarity and interpretation. Furthermore, to provide clearer presentation and contextual interpretation of the SNP-infection associations, we revised the Results section as follows:

Page 5: "Since *GTF3C5* knockdown has been shown to increase endocytosis and internalization of transferrin,[28] and cellular iron uptake influences risk of infection,[29] we investigated the association between rs2905094 and susceptibility to infection using large case-control GWAS of severe malaria[24] and bacteremia[25] (Supplementary Table 9). Since the effect of rs2905094 on transferrin levels was restricted to East Africa, we compared fixed effects meta-analyses of severe malaria GWAS data from East Africa (i.e. Kenya, Malawi, and Tanzania) and West Africa (The Gambia, Mali, Burkina Faso, Ghana, Nigeria, and Cameroon; Supplementary Fig. 7). We found that rs2905094 was associated with 12% (OR=0.88, 95% CI 0.78, 0.98) protection against severe malaria in East Africa where it altered transferrin levels but was not associated with protection in West Africa where it did not alter transferrin levels (OR=0.95; 95% CI 0.87, 1.03; Fig.

3e and Supplementary Fig. 7). rs2905094 was also associated with 15% (OR=0.85, 95% CI 0.75, 0.97; P=0.02) protection against overall bacteremia in Kenyan children (Fig. 3e).”

Page 6: “Since hepcidin is implicated in the pathophysiology of malaria and bacterial infections in mouse models,[12,13,32] we determined the association between rs73596248 and these infections in the case-control GWAS of severe malaria and bacteremia described above.[24,25] We found that rs73596248 was associated with protection against bacteremia due to *Klebsiella pneumoniae* (OR=0.22; 95% CI 0.05, 0.92; P=0.04) and *Staphylococcus aureus* (OR=0.56; 95% CI 0.33, 0.92; P=0.02). Protection against *Escherichia coli* and overall bacteremia did not reach statistical significance, while severe malaria was not associated with the rs73596248 variant (Fig. 4c).”

Page 6: “The Dantu blood group locus, marked by variants associated with soluble transferrin receptor (sTfR) levels, showed strong and concordant protective effects against both bacteremia and severe malaria. The lead sTfR GWAS SNP, rs552439837, was 20% protective against bacteremia (OR=0.80; 95% CI 0.68, 0.95; P=0.01; Fig. 5c), as previously shown using rs186873296.[34] Since rs552439837 was missing in the case-control study of severe malaria, we identified a tag SNP, rs141274959, from among the genome-wide significant sTfR GWAS SNPs (Supplementary Table 11). rs141274959 is in strong LD with rs552439837 (r^2=0.9) and was highly protective against severe malaria (OR=0.56; 95% CI 0.46, 0.69; P=3.3x10^{-9}).”

Data and methodology
As mentioned above, I think the methods for Discovery and Replication GWAS seem appropriate although I have some concerns about the how the heterogeneity of contributing study designs and I and the resulting differences and gaps in the data collected. For example that transferrin was not measured in two sites and why α1-Antichymotrypsin being used as a marker of inflammation in one of the studies, rather than a more standard marker such as CRP.

Response: We acknowledge that heterogeneity in study design and biomarker availability is a limitation of multi-site analyses. Transferrin was measured in all sites, however transferrin saturation (TSAT) could not be calculated in Uganda and South Africa because serum iron was unavailable, as blood samples had been collected in EDTA vacutainers, which chelated iron. In The Gambia, α1-Antichymotrypsin (ACT) was used instead of CRP, which was not available in the samples. ACT is a validated acute-phase reactant that correlates strongly with CRP, rising quickly like CRP but remaining elevated for longer, making it a reliable alternative marker of inflammation. To minimize the impact of site-specific heterogeneity, we conducted all analyses for each site, then combined by meta-analysis. We have now clarified in the Discussion that differences in biomarker availability across sites are a limitation of the study, as follows:

Page 9: “A potential limitation is heterogeneity in the availability of iron biomarker and red cell indices data across sites. For example, TSAT could not be calculated in Uganda and South Africa due to missing serum iron measurements, and ACT was used instead of CRP in The Gambia. To minimize bias, all analyses were conducted within sites and combined by meta-analysis.”

I also think it would have been useful to show red cell indices, other than haemaglobin, as more clinically relevant reflection of iron status than just the biochemical markers presented.

Response: We agree that red cell indices such as mean corpuscular volume (MCV) and mean corpuscular hemoglobin concentration (MCHC) provide clinically relevant measures of iron status. However, the main iron biomarkers used in our analyses (transferrin, ferritin, hepcidin, sTfR, serum iron, and TSAT) remain well-established indicators of iron homeostasis that have been used in genome-wide association analyses in European populations. We have now incorporated available MCV and MCHC data into our analyses (MCV: 2,237 children across Kenya, Uganda, and The Gambia; MCHC: 1,374 in Kenyan and Ugandan children) and updated Figure 3d, Supplementary Table 1, Supplementary Figure 8, and the Methods and Results sections accordingly.

Page 5: "Associations between rs73596248 and other individual iron biomarkers and red cell indices did not reach statistical significance (Supplementary Fig. 9a)."

Page 21: "Red cell indices data included hemoglobin levels, Mean Corpuscular Volume (MCV), and Mean Corpuscular Hemoglobin Concentration (MCHC), measured using automated blood analyzers (Beckman Coulter)."

For the study looking for an association between the SNPs and severe malaria and bacteraemia I there was less description of the methods and the data making it difficult to comment on. Providing more information including a descriptive table of the population characteristics would be helpful.

Response: We agree that the initial version of the manuscript included limited methodological detail regarding the analyses of SNP associations with severe malaria and bacteremia. We have now expanded the Methods section to describe these analyses in greater detail, including information on the severe malaria and bacteremia GWAS datasets, case–control sample sizes, analytical approach, and power calculations. To improve transparency and allow clearer interpretation, we have also added Supplementary Table 9, which summarizes the sample characteristics of the infection GWAS datasets by identified African-specific iron-related lead variants. These additions provide a more complete description of the data and analytical framework for the SNP–infection analyses.

I was specifically interested to understand when these samples had been taken given the changing malaria epidemiology and had to go to other publications to find this. I was surprised to learn that that the original Malariagen study was conducted in Kilifi (conducted 1995-2008). Indeed a recently published study by the group designed specifically to explore the relationship between the Dantu and bacteriaemia, analysed separately data from 1998-2003, a period of high transmission and data from 2003-2010, a period of low transmission. They reported that the protective effect of Dantu against all-cause bacteremia was only significant during the high malaria transmission period suggesting that the protective effect was most likely mediated indirectly by its protective effect against malaria. It is not clear why a similar approach was not taken in this current study and somewhat contradicts which the authors state "….the Dantu NE blood group phenotype, is under strong selective pressure for protection against malaria along the Kenyan coast." (Line 255).

Response: We agree that malaria epidemiology in Kilifi has changed substantially over time and appreciate the importance of considering transmission intensity when interpreting genetic associations. The iron and inflammatory biomarkers in our study were measured from archived plasma samples collected during annual cross-sectional surveys between 1998 and 2016, spanning both high (pre-2003) and subsequent lower malaria transmission periods. As the reviewer notes, our colleagues have already demonstrated, using overlapping sample sets, that the protective effect of the Dantu NE blood group against all-cause bacteremia is most likely mediated indirectly through protection against malaria during periods of high transmission. The lead SNP reported in that study is in strong linkage disequilibrium ($r^2 > 0.9$) with our lead SNP at this locus, and we therefore did not repeat the same stratified analyses here. Regarding our statement that the Dantu NE blood group is under strong selective pressure for protection against malaria along the Kenyan coast, we intended this as a reference to prior population-genetic and epidemiologic studies, rather than a conclusion from the present dataset. To avoid any ambiguity, we have clarified these points in the Results and Discussion sections as follows:

Page 6, Results: "A complex structural variant at this locus, termed DUP4, encoding the Dantu NE blood group phenotype, has previously been shown to be under strong selective pressure due to its protective effect against malaria along the Kenyan coast.[27,33] The lead sTfR SNP in our GWAS, rs552439837, was in strong LD with DUP4 (e.g. r^2>0.9 with the previously identified tag SNP, rs186873296),[33] which was also associated with increased sTfR levels ($P=1.3x10^{-6}$)."

Page 8, Discussion: "Recent analyses from the same population showed that another SNP tagging the Dantu polymorphism, rs186873296, and in strong LD with our lead SNP (r^2>0.9), is associated with protection against bacteremia via its effect on malaria risk.[34]"

We have also explicitly stated in the Methods the timeframe of biomarker collection.

Page 19: "Iron and inflammatory markers were measured from blood samples collected at annual cross-sectional surveys (between 1998 and 2016) based on the availability of plasma samples archived at -80^{0}C."

Analytical approach

In the section of statistical analysis section (line 764-814), there is no description of how the data were analysed for the association between SNPs and bacteriaemia.

Response: We agree that our description of the SNP–bacteremia analysis was too brief in the original version. We have now clarified these analyses in the Methods section as follows:

Page 23: "For SNP-bacteremia associations, we identified our African-specific SNPs in the largest GWAS of bacteremia in African populations,[25] merged the genotypes with the hospital clinical data, and conducted logistic regression analyses adjusting for age, sex, and principal components."

In the section on sample size and power (line 840-846), there is no description of the sample

size and power calculations for the studies looking for association of SNPs with severe malaria and bacteriemia.

Response: We have now added power estimates for the association of SNPs with severe malaria and bacteraemia in the Methods section. The associations between SNPs and severe malaria and bacteriemia were targeted follow-up of lead variants rather than new genome-wide scans, and therefore formal a priori power calculations were not undertaken as for the discovery GWAS.

Page 24: "For the association between lead SNPs and severe malaria or bacteraemia, we estimated that the severe malaria dataset (7,957 cases, 7,746 controls) had ~80% power to detect an odds ratio ≥1.15 for a variant with MAF = 5% at α = 0.05, while the bacteremia dataset (1,970 cases, 4013 controls) had ~80% power to detect an odds ratio ≥1.40."

Clarity and context
I this paper would benefit from being written more clearly, coherently and concisely. This would make it both stronger and more accessible. There are important details missing and very limited mention of any study limitations which I think are essential to address. I think it would also be helpful to clarify what's new and significant in terms of implications for clinical practice or public health. Perhaps one way to make this clearer would be to give an example of how results of GWAS studies of iron status in other (eg European populations) have impacted on clinical care or outcomes?

Response: We have carefully edited the text throughout the manuscript for improved clarity, conciseness, and logical flow. We have expanded the Methods and Discussion to include additional details that were previously missing. For example, we have provided more details on the iron-infection studies and analyses and included additional comprehensive analyses of selective pressure, statistical fine-mapping, and haplotype level-associations. We now also provide a more comprehensive description of study limitations including heterogeneity in biomarker availability across sites, and limited power to detect small-effect variants.

To strengthen the clinical and public-health context, we have clarified what is new and significant about our findings in the Discussion, particularly the identification of African-specific loci influencing iron status and infection susceptibility, and how these results contribute to understanding iron regulation in diverse populations. We have also added a relevant example illustrating how large European iron-GWAS have been foundational for several Mendelian randomization studies that clarified causal relationships relevant to clinical guidelines.

Page 10: "we identify African-specific genetic loci that influence iron biomarkers and risk of severe malaria and bacteremia. These findings have important implications for understanding genetic and environmental interactions shaping iron regulation and infection risk in African populations. While large European iron-GWAS have been foundational for several Mendelian randomization studies that clarified causal relationships relevant to clinical guidelines,[45,46] our results highlight the need for Africa-centric genomic data since genetic risk scores and instrumental variables identified in European populations were poorly predictive in African populations. Africa-specific

data is needed to guide diagnostic screening and inform targeted interventions and equitable global health strategies. Our findings have potential public health relevance for iron supplementation programs since genetic differences in iron regulation may modify the balance between correcting deficiency and increasing infection risk, underscoring the importance of population-specific approaches. For instance, routine or universal iron supplementation or fortification, especially in the context of elevated hepcidin, may lead to iron accumulation in the gut or dysregulated iron distribution, potentially increasing susceptibility to enteric and systemic infections.[8,47] Future studies should explore the mechanistic pathways linking the identified variants to iron-pathogen interactions to inform safe and targeted interventions in African settings."

We believe these revisions have substantially improved the clarity, completeness, and accessibility of the manuscript.

Reviewer #2 (Remarks to the Author)
I appreciate the authors' efforts to address my comments. However, I still have some concerns.

I appreciate the authors included the statement that the frequency of rs2905094-T was 9 % in Burkina Faso. Nonetheless, they refer to this as a "low frequency." Generally, "low frequency" denotes frequencies of 5 % or less, often even below 1 %. A 9 % frequency would be considered common rather than low.

The authors claim that they cannot publish the haplotype configuration for ethical reasons. I am not requesting individual-level data; it would be sufficient to provide a representative configuration and its count. The data in SFig 4 are too vague. For example, the manuscript states that Hap1 is present at less than 20 % in South Africa and Hap2 at about 15 %. Yet, looking at the third row from the top in SFig 4, Hap1 appears to be more than twice as frequent as Hap2, which is inconsistent with the manuscript. Displaying only the representative SNP alleles that define each haplotype and presenting them in a table with numeric values would resolve this. What matters most is identifying which haplotype carries the causal variant. Differences in haplotype configuration between EUR and other populations would also be clearer if shown using the representative alleles and their counts.

Response: We thank the reviewer for their careful re-examination of the haplotype frequencies and for highlighting the inconsistency between the manuscript text and SFig 4. Upon re-checking the South African data, we confirm that the reviewer is correct: Haplotype 1 is present at 31.3% and Haplotype 2 at 14.7% in South Africa. We have corrected the text to reflect these values and now report exact haplotype counts and frequencies in a new Supplementary Table 6.

Supplementary Table 6. Distribution of haplotypes carrying the rs2905094-T allele at the *GTF3C5* locus across African populations

Population	Haplotype 1, n (%)	Haplotype 2, n (%)	Other n (%)
Kenya	109 (32.2)	113 (33.3)	117 (34.5)
Uganda	115 (23.0)	100 (20.0)	285 (57.0)
South Africa	66 (31.3)	31 (14.7)	114 (54.0)
Burkina Faso	61 (52.1)	10 (8.6)	46 (39.3)
The Gambia	151 (62.1)	0 (0)	92 (37.9)

We have also rewritten the results section as follows:

Page 4: "Haplotype 1 predominated in West Africa (The Gambia 62%, Burkina Faso 52%) but was less frequent in East and Southern Africa (Kenya 32%, Uganda 23%, South Africa 31%), whereas Haplotype 2 was absent in The Gambia, present at 9% in Burkina Faso and 15% in South Africa, and observed at moderate frequencies in East Africa (Kenya 33%, Uganda 20%; Supplementary Table 6). However, in haplotype-level analyses in East Africans, both Haplotypes 1 and 2 were associated with lower transferrin levels, and these effects disappeared after conditioning on rs2905094, indicating that the signal is driven by rs2905094 or an unidentified variant in strong LD with rs2905094 rather than by a haplotype-specific effect (Supplementary Table 7)."

From the haplotype association results, the hypothesis that the association observed in these populations is driven by Hap2 is not supported.

Summarizing the findings thus far: rs2905094 is significantly associated with transferrin levels only in East African populations comprising Kenya and Uganda. In the fine-mapping of this group, rs2905094 has the highest PIP of about 0.57. The rs2905094-T allele is common in East Africa, Other Africa, and Europe. The data do not support the notion that a causal variant lies on a specific haplotype present only in East Africans and absent in other groups.

The explanation for the lack of association with rs2905094 outside East Africa remains insufficient. We cannot rule out issues related to quality control or population structure, and the absence of association in other groups suggests that the signal was not replicated. The tone regarding the association of this SNP rs2905094 should be moderated.

Response: Reexamining our data, we agree with the reviewer that the haplotype results do not support the hypothesis that the observed association is driven by a unique haplotype present only in East African populations. We have revised the manuscript to remove references suggesting a Haplotype 2-specific causal mechanism.

We agree that the explanation for the lack of association outside East Africa remains unresolved. We have moderated the tone in the manuscript to acknowledge that alternative explanations including differences in environmental context or gene-environment interactions, linkage disequilibrium, and population or complex genomic structure, may contribute to the observed specificity in East African populations, and that a population-specific causal variant cannot be inferred from our data. We think it is unlikely that issues with quality control would explain a specific association in two separate East African populations, but not in West and South Africa especially as the genotypes have been used to identify associations in other published studies (example, https://www.nature.com/articles/s41591-024-02944-5). Other studies have similarly demonstrated substantial genetic heterogeneity among populations from East, West, and South Africa (example, https://www.nature.com/articles/s41467-025-58789-0).

We have revised the Discussion as follows:

Page 7-8: "Although the effect allele at the *GTF3C5* locus is common across African ancestry populations, and even more frequent in Europeans (33%), its association with transferrin levels was observed only in the East African cohorts. To investigate this geographic specificity, we examined local haplotype structure and found that rs2905094 occurs on multiple haplotypic backgrounds that differ in frequency across East and West Africa. Haplotype-level association analyses did not support a haplotype-specific effect suggesting that the observed association is not driven by a haplotype uniquely present in East Africa. Fine-mapping analyses identified rs2905094 as the variant with the highest posterior inclusion probability, although the possibility of a correlated variant in linkage disequilibrium cannot be excluded. Nonetheless, the population-specific nature of the association remains incompletely explained and may reflect differences in linkage disequilibrium, population structure, environmental context or gene-environment interactions, and unmodeled confounding, underscoring the importance of examining regional genomic diversity in African populations.[19,23]"

We believe these revisions improve the accuracy and balance of the interpretation and directly address the reviewer's concerns.
